# A novel synthetic chemistry approach to linkage-specific ubiquitin conjugation†
†Electronic supplementary information (ESI) available: Full experimental details are provided. See DOI: 10.1039/c5ob00130g



**DOI:** 10.1039/c5ob00130g

**Published:** 2015-03-04

**Authors:** Rachel E. Morgan, Vijay Chudasama, Paul Moody, Mark E. B. Smith, Stephen Caddick

**Affiliations:** a Department of Chemistry , University College London , 20 Gordon Street , London , UK . Email: VPEnterprise@ucl.ac.uk ; Fax: +44 (0)20 7679 7463 ; Tel: +44 (0)20 3108 5071

## Abstract

Ubiquitination is of great importance as the post-translational modification of proteins with ubiquitin, or ubiquitin chains, facilitates a number of vital cellular processes. Herein we present a facile method of preparing various ubiquitin conjugates under mild conditions using michael acceptors based on dibromo-maleimides and dibromo-pyridazinediones.

## Abstract 2

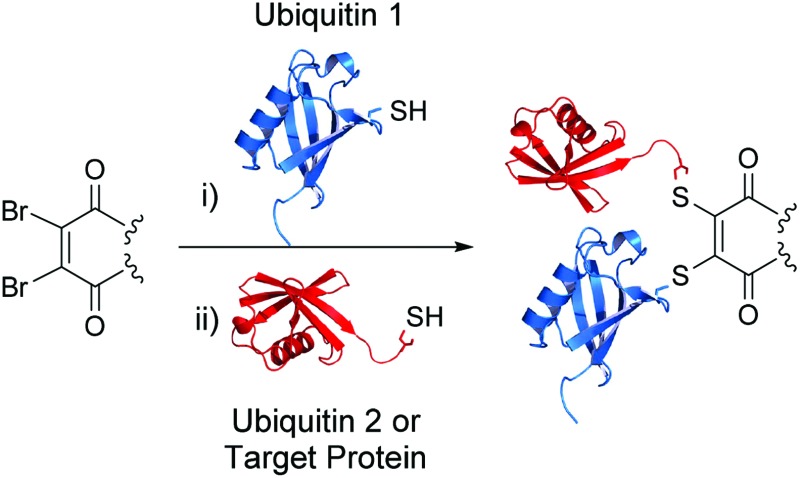
Site-specific ubiquitin cysteine mutants enable an elegant method for the linkage-specific conjugation of ubiquitins through dibromomaleimides and dibromopyrdazinediones.

## 

Ubiquitin is a small, highly conserved protein (∼8.5 kDa) that is attached to target proteins through a post-translational modification process known as ubiquitination. Ubiquitination has a significant role in a number of cellular processes^1^ and the full extent of its importance is, as yet, not fully realised. Methods that facilitate the study of ubiquitination have the potential to significantly impact this fascinating and rapidly expanding area of research.^2^ In the process of ubiquitination, ubiquitin can be added to the target protein singly or in the form of chains. These linkages to and between ubiquitins involves three enzymes: E1, E2 and E3. These enzymes generate an isopeptide link between the C-terminal glycine (G76) of a ubiquitin and a lysine residue on a second ubiquitin or a target protein. There are seven lysines residues on a ubiquitin through which the isopeptide link can form: K6, K11, K27, K29, K33, K48 and K63. The selectivity inherent in the requirement of three enzymes for ubiquitin conjugation, in particular E3, makes accessing significant quantities of a range of ubiquitinated proteins a major challenge. Synthetic efforts have sort to mimic the activity of these enzymes by conjugating ubiquitins together using synthetic techniques.^3^

When considering techniques to conjugate target proteins to ubiquitin, ideally they should be easily accessible, mild and require minimal steps. Otherwise the techniques would preclude the modification of proteins of interest that are not amenable to high temperatures or unusual buffer systems. We sought to build upon the existing chemistry in our laboratory to develop a novel, accessible and mild method of generating ubiquitin conjugates.^4,5^

We have recently shown dibromomaleimide **1** and dibromopyridazinedione **2** (see Fig. 1) to be highly effective moities for the modification of proteins, through addition to cysteines, under mild conditions. Conjugation of various chemical entities, such as thioglucose and glutathione, onto proteins has been readily achieved.^4,5^

**Fig. 1:**
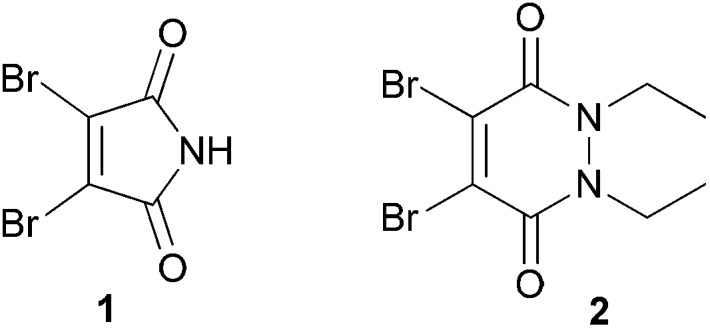
Structures of selective cysteine modification reagents: dibromomaleimide **1** and dibromopyridazinedione **2**.

Ubiquitin does not contain any cysteine residues, which makes chemical modification of a ubiquitin cysteine mutant *via* synthetic chemistry an attractive prospect. We envisaged a method involving the coupling of two ubiquitin cysteine mutants *via* a maleimide or pyridazinedione in place of the isopeptide bond. This could be achieved by cysteine mutation of a target lysine on one ubiquitin and a terminal glycine on another ubiquitin (G76). Moreover, these cysteine mutants could be expressed using standard expression systems, thus allowing for all the advantages that this provides, such as cost, time and scalability.

Therefore the following strategy was devised for the construction of ubiquitin conjugates (see Fig. 2). Firstly, a lysine to cysteine ubiquitin mutant, UbKXC **3**, was to be reacted with dibromomaleimide **1** or dibromopyridazinedione **2** to form functionalised ubiquitin **5**. Reaction of this species with glycine to cysteine ubiquitin mutant UbG76C **6** should then provide access to linkage-specific ubiquitin–ubiquitin conjugate **7**.

**Fig. 2:**
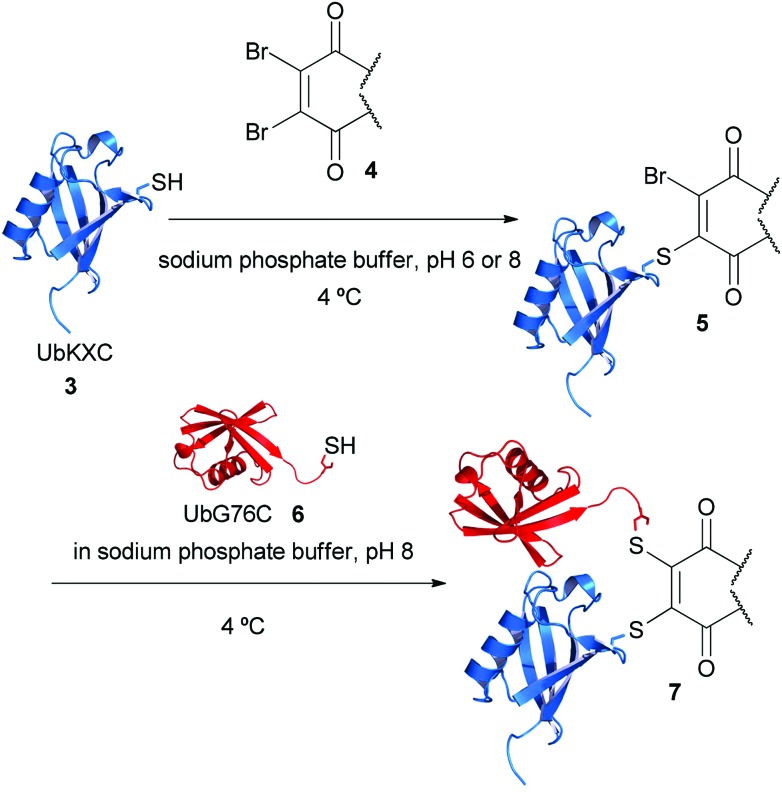
Overall strategy for the formation of ubiquitin conjugates. Structure modified from PDB ID: 1UBQ.

To this end, we generated a range of ubiquitin cysteine mutants: UbK27C, UbK48C, UbK63C and UbG76C. Of particular interest are ubiquitin conjugates attached through the K48 and K63 positions which have been indentified in a number of biological processes.^6^ Lysine 27 is noted as one of the most challenging to modify using chemical techniques and would therefore offer a particular challenge to any new methodology.^7^ Also, in order to examine the feasibility of using the approach to affect conjugation of ubiquitin onto a second protein, we generated green fluorescent protein (GFP) mutant GFPS147C^8^ which we could then use to generate ubiquitin-GFP conjugates.

Ubiquitin mutants UbK27C, UbK48C and UbK63C (100 μL and 1 mg mL^–1^) were each incubated with 5 equivalents of dibromomaleimide **1**, on ice for 1 h. Consistent with our previous experience of protein modification with dibromomaleimides (*e.g.* fast reaction time, exceptional thiol selectivity), complete modification was observed.^4^ In order to reduce the potential for hydrolysis of the maleimide motif this modification was carried out in sodium phosphate buffer at pH 6 (50 mM sodium phosphate pH 6, 75 mM NaCl). Incubation of dibromomaleimide **1** with WT ubiquitin afforded no reaction after 24 h, indicating that the observed modifications had occurred on the cystienes that had been introduced by site-directed mutagenesis.

After removal of the excess dibromomaleimide from the reaction mixture by ultrafiltration, UbG76C mutant **6** was added to each of the samples. UbG76C was added in an equal volume of sodium phosphate buffer at pH 8 (50 mM soduim phosphate pH 8, 75 mM NaCl) in order to increase the rate of coupling. Gratifyingly, for conjugates containing UbK48C and UbK63C, coupling was complete after only 1 h on ice (Fig. 3). Although a longer incubation period was required for UbK27C, the product was observed after 24 h, this observation is consistent with the difficulties reported in the literature and may reflect the reduced accesibility of this residue.^7^

**Fig. 3:**
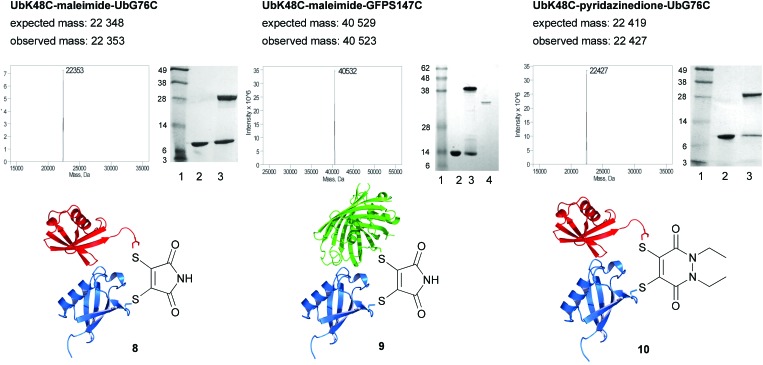
Conjugation reactions for the formation of bis-protein conjugates **8–10**. Structures modified from PDB ID: ; 1UBQ and ; 2B3P. Lanes in SDS-PAGE gels are: 1-SeeBlue Plus2 ladder, 2-UbK48C, 3-coupling reaction, 4-GFPS147C. The band at ∼11 kDa in lane 3 is due to excess ubiquitin.

We next set about evaluating the coupling of UbK48C and UbG76C on a larger scale (600 μL at 1 mg mL^–1^). This enabled us to show that purification using size exclusion enables clean separation of the coupled ubiquitin dimer from the uncoupled ubiquitin and to obtain an excellent representative yield, 78% (see ESI† for details).

Our coupling technique was next used to generate ubiquitin-GFP conjugates. Both maleimide modified UbK48C and UbG76C were incubated with GFPS147C. Conjugation was successful for both conjugates and the coupling had no adverse effect on GFP fluorescence (see ESI† for details), thus indicating the potential for the use of this method to conjugate ubiquitin to a target protein.

We next evaluated dibromopyridazinedione **2** as a tool for conjugation. The complete hydrolytic stability of this reagent allowed for all the reactions to be performed at pH 8.^5^ Although it should be mentioned that it is still possible to carry out conjugation reactions at pH 6. Gratifyingly, using the strategy outlined in Fig. 2 at pH 8, di-ubiquitin conjugates UbK48C–UbG76C and UbK63C–UbG76C were generated. Whilst modification of UbK27C with dibromopyridazinedione was achievable, the reduced reactivity of the pyridazinedione modified UbK27C precluded couping with UbG76C under a reasonable time scale. However, to our delight, ubiquitin-GFP conjugates using dibromopyridazinedione **2** and UbK48C and UbG76C were successfully isolated.

In conclusion we have presented a method of generating ubiquitin-protein conjugates in a facile manner which is achieved under mild conditions using short reaction sequences. The use of this method to generate linkage-specific ubiquitin–ubiquitin conjugates and ubiquitin-GFP conjugates demonstrates the potential of this method to provide ready access to a range of desired ubiquitin conjugates. In comparision to other strategies,^3^ this method allows the controlled, linkage-specific coupling of ubiquitins, which can be expressed in *E. coli*, and it does not require protecting groups or the use of non-natural amino acids. We believe that this methodology will be of use to researchers seeking to delineate further the functional consequences of protein ubiquitination.

We gratefully acknowledge UCL, EPSRC and Wellcome Trust for funding. We would also like to thank Dr L. Cabrita and Prof. J. Christodoulou for supplying the original ubiquitin wt clone.
