# Supplementary material for: A novel synthetic chemistry approach to linkage-specific ubiquitin conjugation
Source: Org Biomol Chem. 2015 Mar 4;13(14):4165–8. doi: 10.1039/c5ob00130g (PMC4372856; doi:10.1039/c5ob00130g)
Supplement: Supplementary file 1 [file OB-013-C5OB00130G-s001.pdf]

Electronic Supplementary Material (ESI) for Organic & Biomolecular Chemistry.
This journal is © The Royal Society of Chemistry 2015

Supporting Information

A novel synthetic chemistry approach to ubiquitin conjugation

Rachel E. Morgan, Vijay Chudasama, Paul Moody, Mark E. B. Smith and Stephen Caddick*

Department of Chemistry, University College London, 20 Gordon St, London, UK.

Fax: +44 (0)20 7679 7463; Tel: +44(0)20 3108 5071;

E-mail: VPEnterprise@ucl.ac.uk.

General Experimental

All reagents were purchased from Sigma-Aldrich, unless stated otherwise, and used without any purification. Solvents were purchased from Fisher and used without purification. 4,5-Dibromo-1,2-diethyl-1,2-dihydro-pyridazine-3,6-dione was synthesised as previously reported.[1]

Mass Spectrometry

LC-MS was performed on protein samples using a Thermo Scientific uPLC connected to MSQ Plus Single Quad Detector (SQD). Column: Hypersil Gold C4 1.9m 2.1 x 50 mm. Wavelength: 254 nm. Mobile Phase: 99:1 Water (0.1% formic acid): MeCN (0.1% formic acid) to 1:9 Water (0.1% formic acid): MeCN (0.1% formic acid) gradient over 4 min. Flow Rate: 0.3 mL/min. MS Mode: ES+. Scan Range: m/z = 500-2000. Scan time: 1.5 s. Data obtained in continuum mode. The electrospray source of the MS was operated with a capillary voltage of 3.5 kV and a cone voltage of 50 V. Nitrogen was used as the nebulizer and desolvation gas at a total flow of 600 L/h. Total mass spectra for protein samples were reconstructed from the ion series using the pre-installed ProMass software using default settings for large proteins in m/z range 500-1500.

Cloning

The WT ubiquitin clone was supplied by Dr L. Cabrita and Prof. J. Christodoulou in a pET2b(+) plasmid. The WT ubiquitin was cloned into a pNIC28-Bsa4 vector with y

ligation independent subcloning using the primers listed in Table 1 (ThermoScientific). The ubiquitin G76C mutant was also cloned from the WT ubiquitin pET2b(+) plasmid into a pNIC28-Bsa4 vector (see Table 1 for primers). Ubiquitin mutants UbK27C, UbK48C and UbK63C were generated from the WT pNIC28-Bsa4 vector using the QuikChange site mutagenesis kit (Ailgent Technologies) with the primers shown in Table 1. DNA sequencing confirmed the identity of the constructs and the vectors were heat-shock transformed into *E. coli* BL21 (DE3) cells for expression.

Table 1. Primers used for the generation of Ubiquitin Mutants.

Mutant	Direction	Sequence 5’ to 3’
WT Ub	Forward	TACTTCCAATCCATGCAGATCTTCGTCAAGACG
	Reverse	TATCCACCTTTACTGTCAACCACCACGTAGACGTAAGAC
UbG76C	Forward	TACTTCCAATCCATGCAGATCTTCGTCAAGACG
	Reverse	TATCCACCTTTACTGTTAACAACCACGTAGACGCAAGAC
UbK27C	Forward	CCATCGAAAACGTTTGCGCTAAAATTCAAGAC
	Reverse	GTCTTGAATTTTAGCGCAAACGTTTTCGATGG
UbK48C	Forward	GATATTTGCCGGTTGCCAGCTCGAAGACG
	Reverse	CGTCTTCGAGCTGGCAACCGGCAAATATC
UbK63C	Forward	CAACATTCAGTGCGAGTCGACCTTAC
	Reverse	GTAAGGTCGACTCGCACTGAATGTTG

Protein expression and purification

The following method was used for the expression and purification of all the ubiquitin mutants:

Cells were grown at 37 °C in 1 L of Luria-Bertani media supplemented with Kanamycin (50 μgL^{-1}). Gene expression was induced, once an OD_{600} of 0.6 was reached, by addition of 1 mM isopropyl-β-D-thiogalactopyranoside. The culture was maintained for a further 16 h at 22 °C. Cells were harvested by centrifugation at 4,000 g for 30 min at 4 °C. The cells were resuspended into a lysis buffer (20 mL, 50 mM sodium phosphate buffer, pH 7.4, 250 mM NaCl, 25 mM imidazole, 1 mM TCEP) containing DNAse I (0.2 mg) and two tablets of a cocktail of EDTA-free protease inhibitors (Roche). Cell lysis was achieved through sonication (6 x 30 s

burst with 1 min cooling intervals). The resultant lysate was centrifuged at 35,000 g for 30 min at 4 °C and the supernatant loaded onto a pre-equilibrated HisTrap HP 5 mL column (GE Healthcare) pre-charged with Ni^{2+}. A linear gradient of imidazole, 25 mM to 1 M was applied to elute ubiquitin. Samples were further purified using a Superdex 75 16/60 size exclusion column (GE Healthcare) equilibrated with 50 mM sodium phosphate buffer, pH 7.4, 250 mM NaCl, 1 mM TCEP.

The protein eluted with one peak of a mass of approximately 12 kDa corresponding to a monomer. Fractions containing the protein were pooled, exchanged into a pH 6 buffer (50 mM sodium phosphate buffer pH 6, 75 mM NaCl), using Amicon Ultra devices (Millipore) and concentrated to 1 mg mL^{-1}. In cases where the protein was stored 1 mM TCEP was added to the buffer, removed by ultracentrifugation (using VivaSpin sample concentrators (GE Healthcare, 5,000 MWCO)) prior to the modifications. The purity and identity of the protein was confirmed by mass spectrometry, with a yield of approximately 8 mgL^{-1} of cell culture.

The GFPS147C mutant was expressed and purified as previously described.[2]

UbK27C

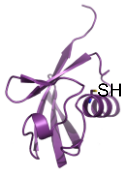

Sequence:

MHHHHHHSSGVDLGTENLYFQSMQIFVKTLTGKTITLEVEPSDTIENVCAKIQ

DKEGIPPDQQRLIFAGKQLEDGRTLSDYNIQKESTLHLVLRLRGG

Expected mass: 11,092

Observed mass: 11,092

(a)

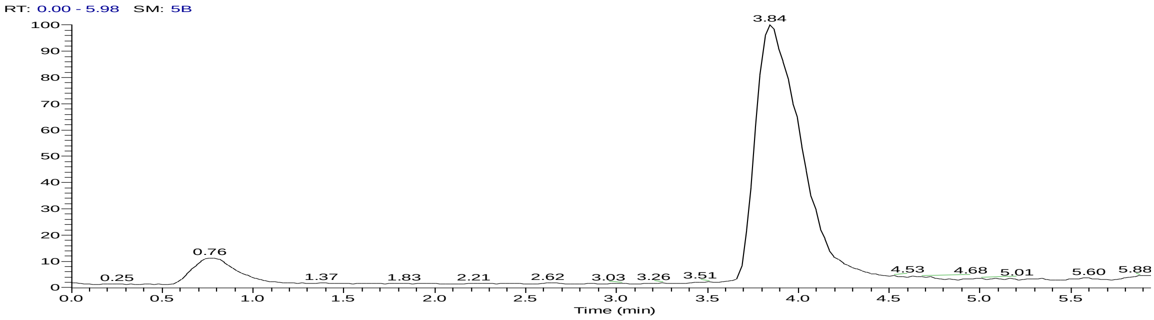

(b)

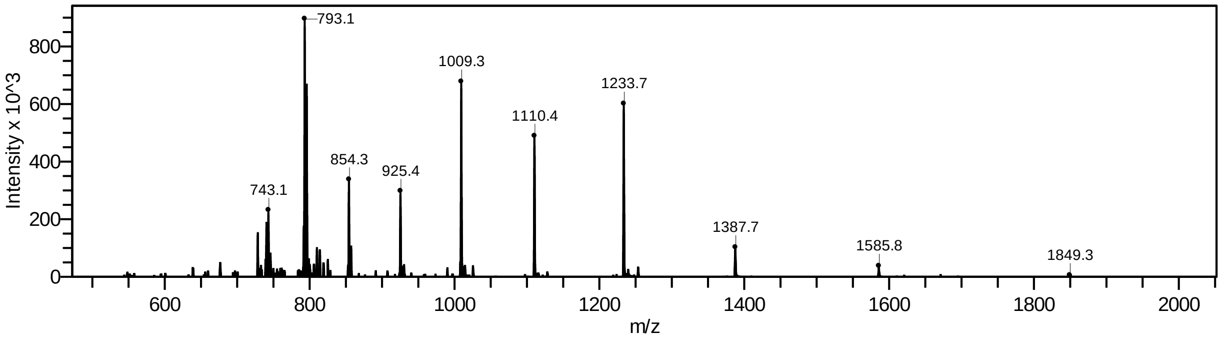

(c)

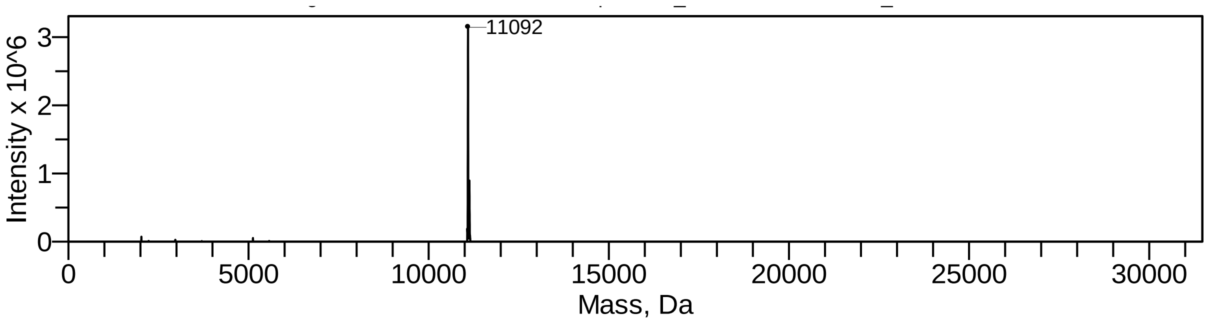

Figure S1: (a) TIC, (b) non-deconvoluted and (c) deconvoluted MS data for UbK27C.

UbK48C

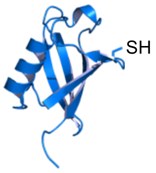

Sequence:

MHHHHHHSSGVDLGTENLYFQSMQIFVKTLTGKTITLEVEPSDTIENVKAKIQ

DKEGIPPDQQRLIFAGCQLEDGRTLSDYNIQKESTLHLVLRLRGG

Expected mass: 11,092

Observed mass: 11,092

(a)

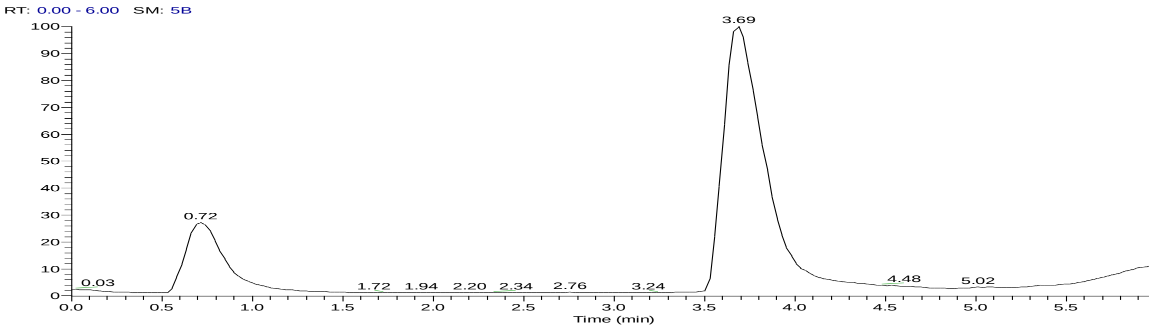

(b)

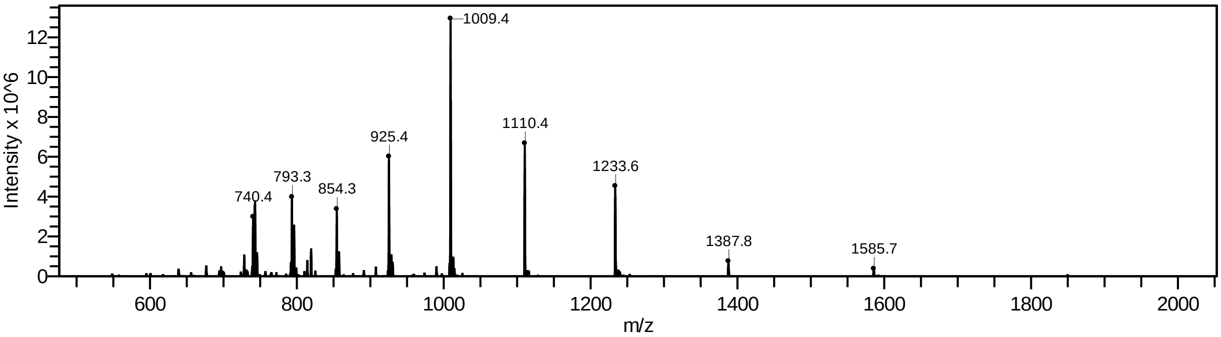

(c)

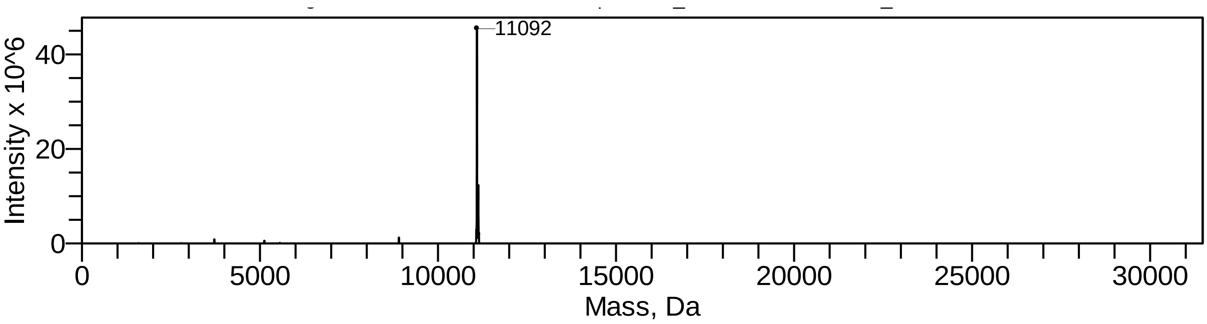

Figure S2: (a) TIC, the first (0.5-1.0 min) peak is the injection peak and does not correspond to a protein ion series, (b) non-deconvoluted and (c) deconvoluted MS data for UbK48C.

UbK63C

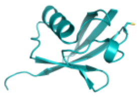

Sequence:

MHHHHHHSSGVDLGTENLYFQSMQIFVKTLTGKTITLEVEPSDTIENVKAKIQ

DKEGIPPDQQRLIFAGKQLEDGRTLSDYNIQCESTLHLVLRLRGG

Expected mass: 11,092

Observed mass: 11,094

(a)

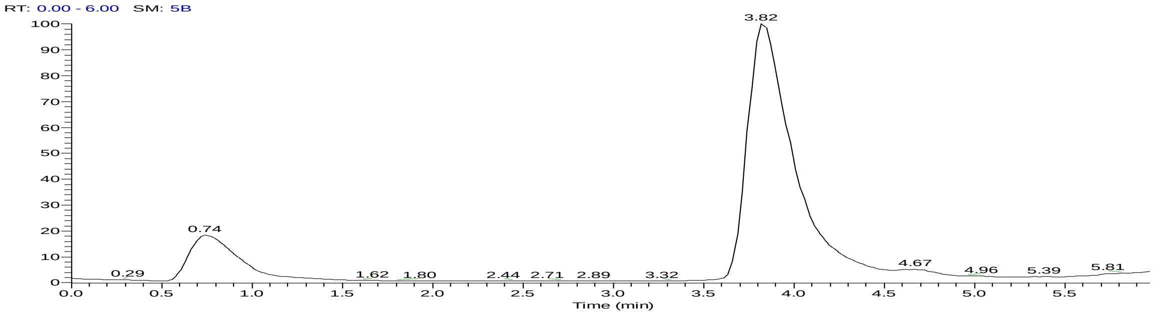

(b)

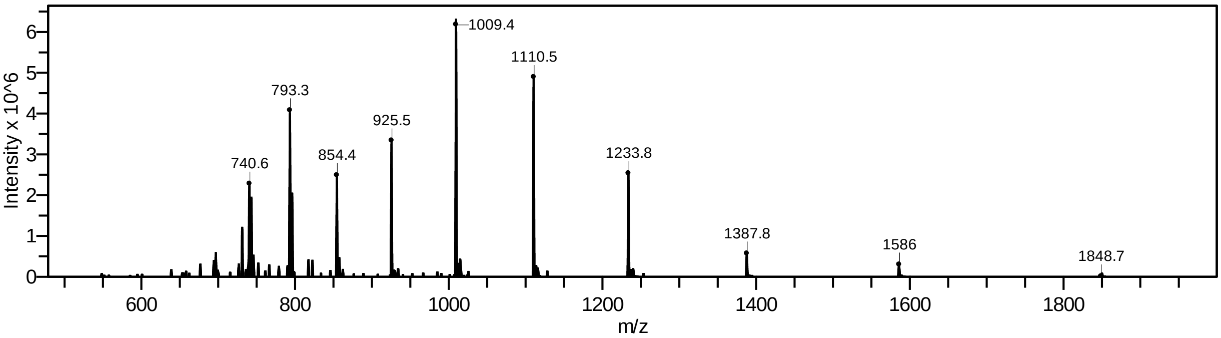

(c)

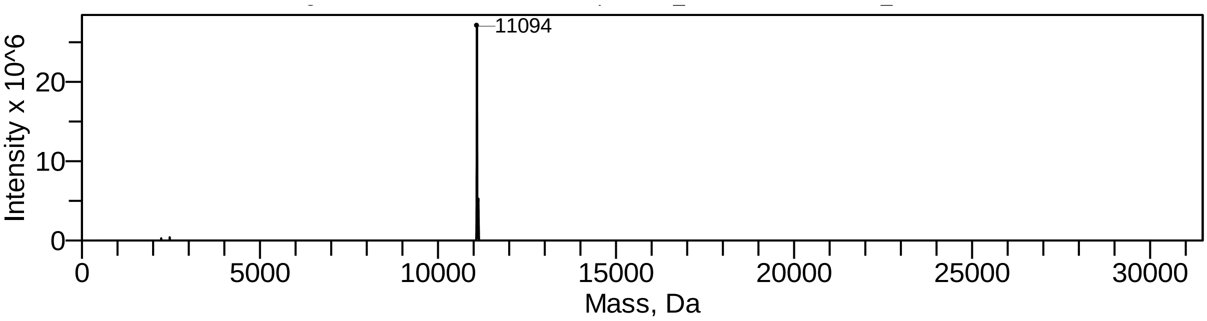

Figure S3: (a) TIC, the first (0.5-1.0 min) peak is the injection peak and does not correspond to a protein ion series, (b) non-deconvoluted and (c) deconvoluted MS data for UbK63C.

UbG76C

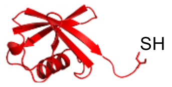

Sequence:

MHHHHHHSSGVDLGTENLYFQSMQIFVKTLTGKTITLEVEPSDTIENVKAKIQ

DKEGIPPDQQRLIFAGKQLEDGRTLSDYNIQKESTLHLVLRLRGC

Expected mass: 11,163

Observed mass: 11,163

(a)

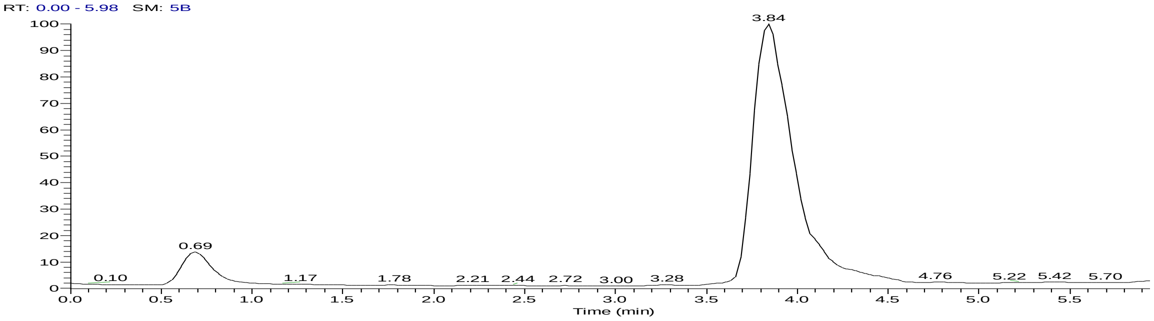

(b)

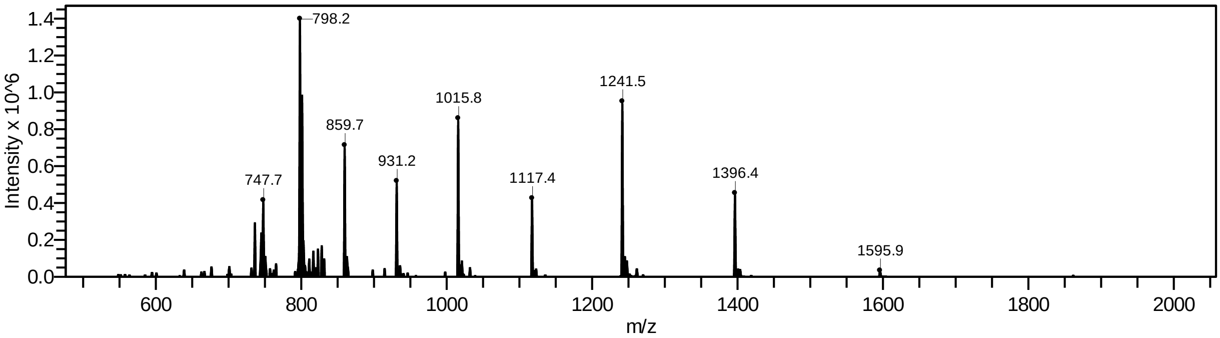

(c)

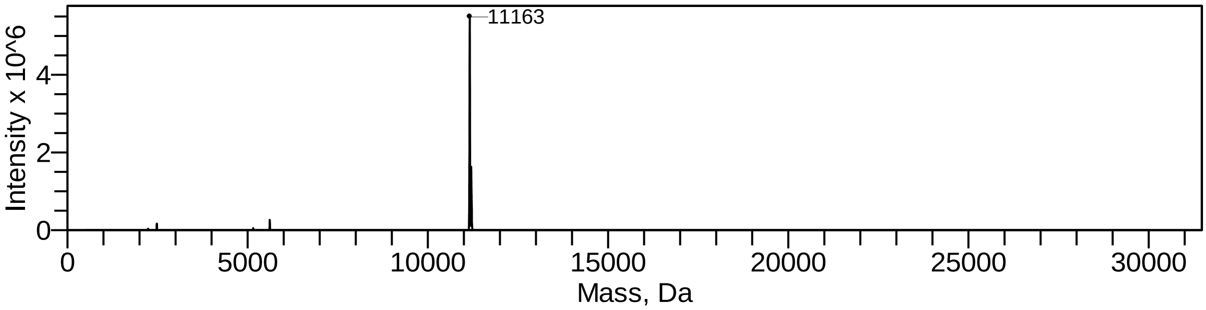

Figure S4: (a) TIC, the first (0.5-1.0 min) peak is the injection peak and does not correspond to a protein ion series, (b) non-deconvoluted and (c) deconvoluted MS data for UbG76C.

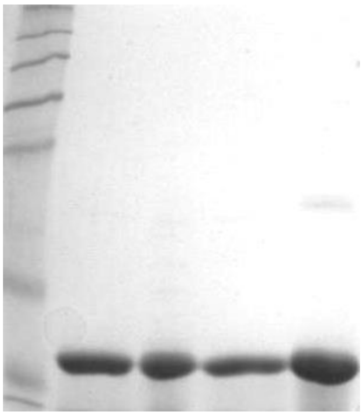

Lane: 1 2 3 4 5

Figure S5: SDS page gel of ubiquitin cysteine mutants.

Lane: 1 – Seeblue Plus2 ladder (Invitrogen); 2 – UbK27C; 3- UbK48C; 4 – UbK63C; 5 – UbG76C. 16% SDS page gel under non-reducing conditions visualised with Coomassie staining.

Maleimide modified ubiquitin

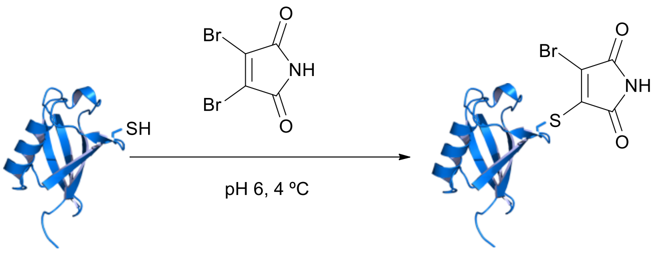

Modification of all cysteine ubiquitin mutants with dibromomaleimide was achieved using the following method. To a solution of cysteine ubiquitin mutant UbXXC (1 mg mL^{-1}, 100 μL) in sodium phosphate buffer pH 6 (50 mM sodium phosphate, 75 mM NaCl, pH 6), dibromomaleimide (5 μL, 9 mM solution in DMF) was added. This reaction mixture was incubated on ice for 1 h. Analysis using LC-MS showed complete modification of the cysteine ubiquitin mutant.

UbK27C-bromomaleimide

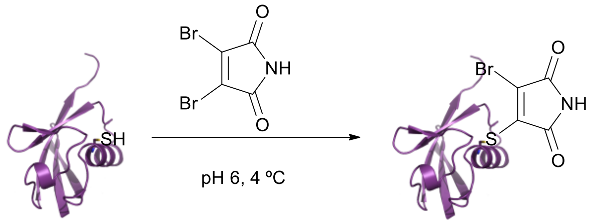

Expected mass: 11,265

Observed mass: 11,267

(a)

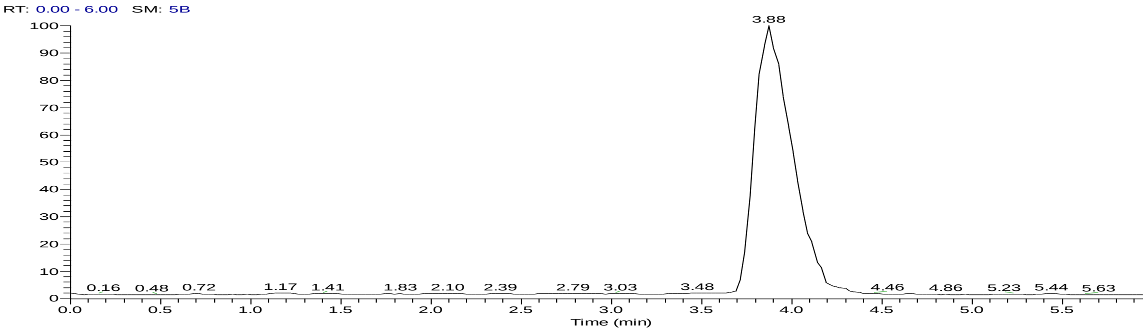

(b)

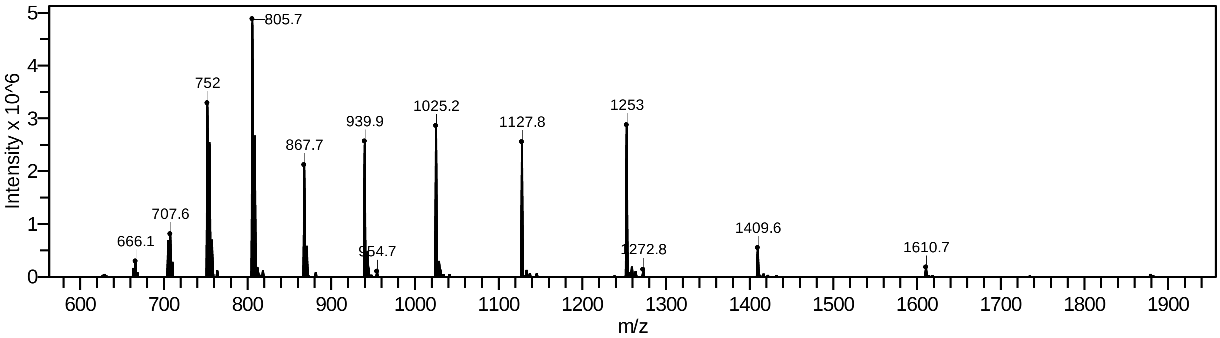

(c)

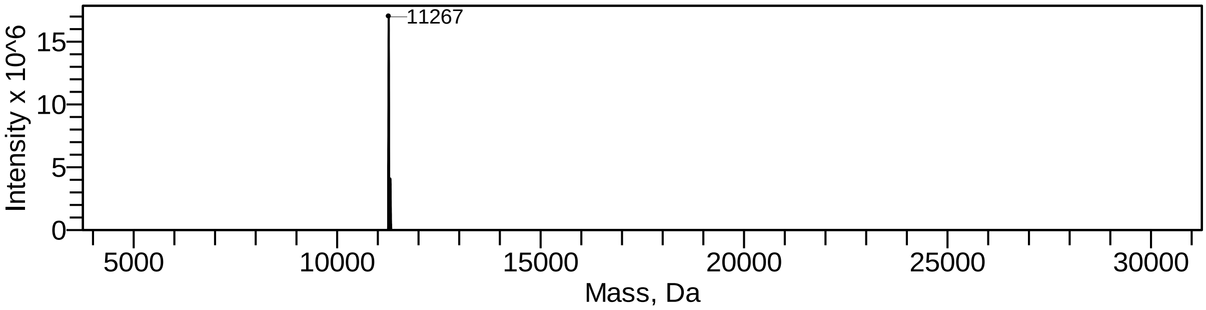

Figure S6: (a) TIC, (b) non-deconvoluted and (c) deconvoluted MS data for UbK27C-bromomaleimide.

UbK48C-bromomaleimide

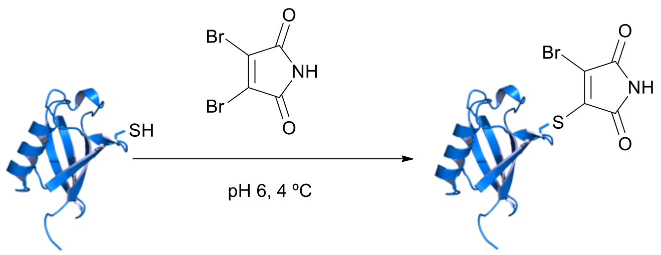

Expected mass: 11,265

Observed mass: 11,267

(a)

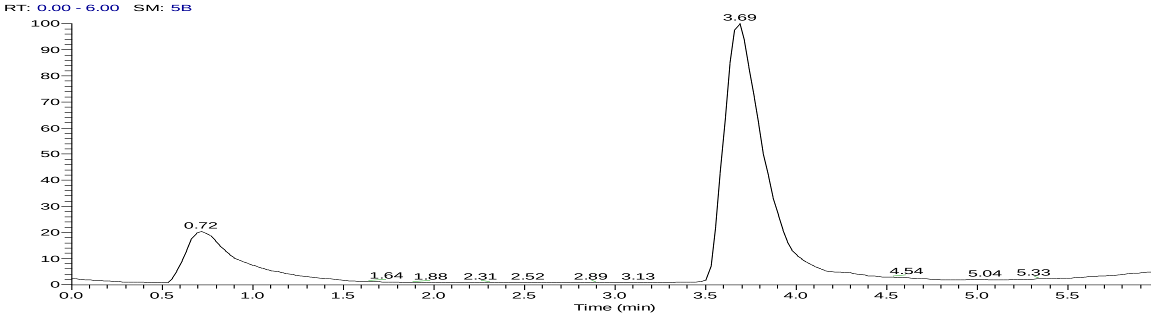

(b)

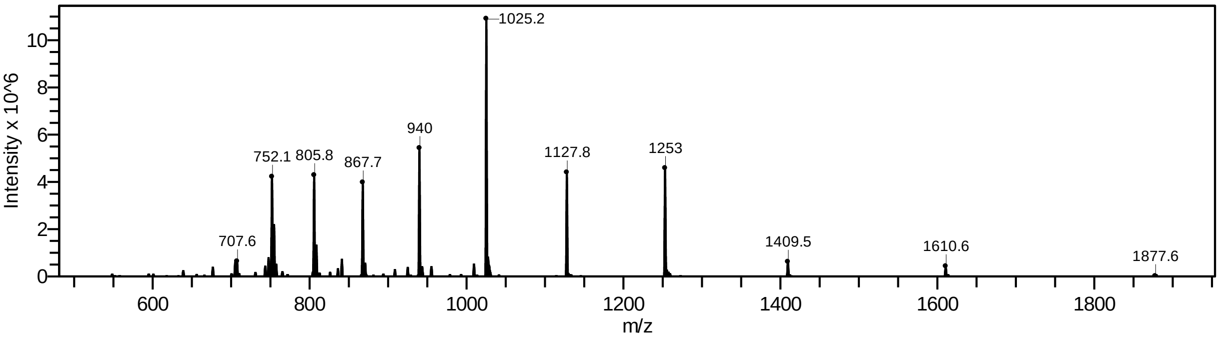

(c)

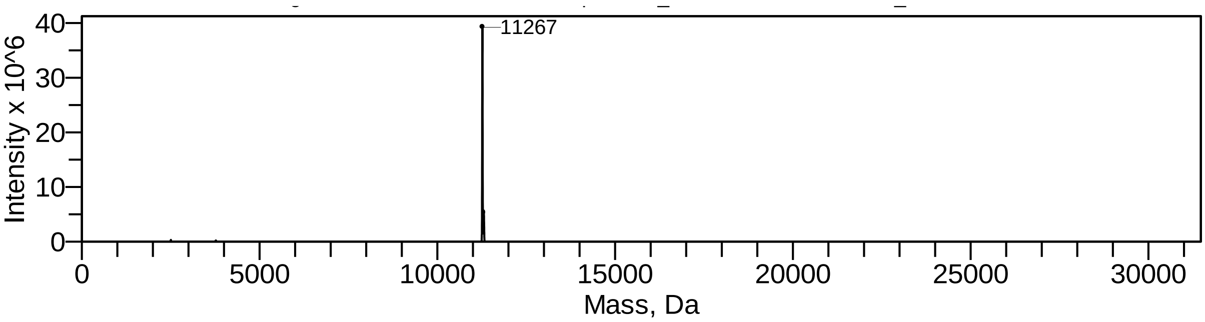

Figure S7: (a) TIC, the first (0.5-1.0 min) peak is the injection peak and does not correspond to a protein ion series, (b) non-deconvoluted and (c) deconvoluted MS data for UbK48C-bromomaleimide.

UbK63C-bromomaleimide

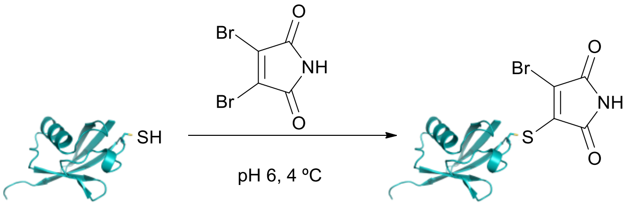

Expected mass: 11,265

Observed mass: 11,267

(a)

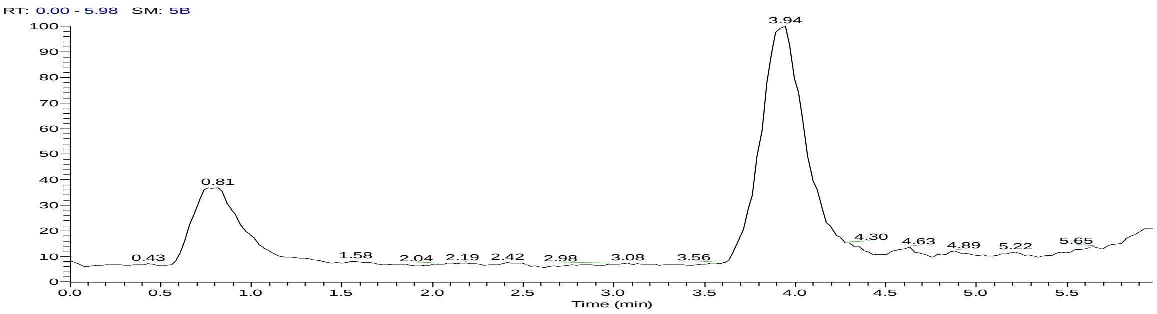

(b)

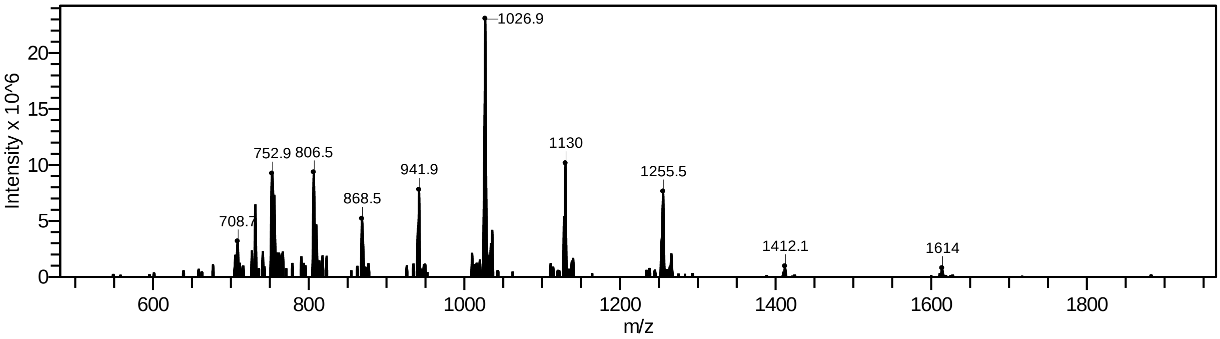

(c)

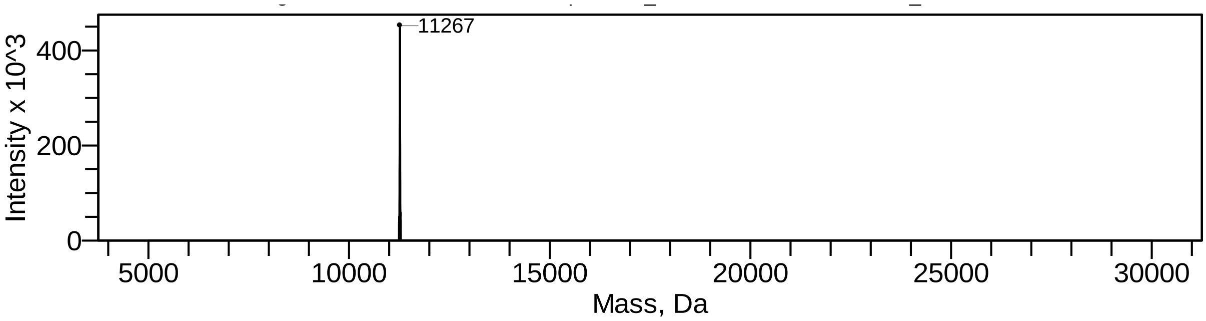

Figure S8: (a) TIC, the first (0.5-1.0 min) peak is the injection peak and does not correspond to a protein ion series, (b) non-deconvoluted and (c) deconvoluted MS data for UbK63C-bromomaleimide.

UbG76C-bromomaleimide

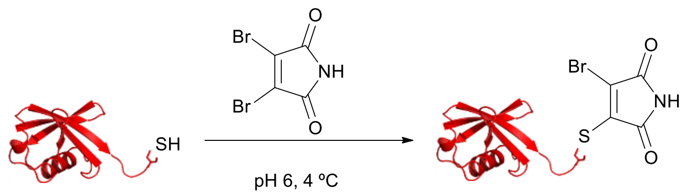

Expected mass: 11,336

Observed mass: 11,336

(a)

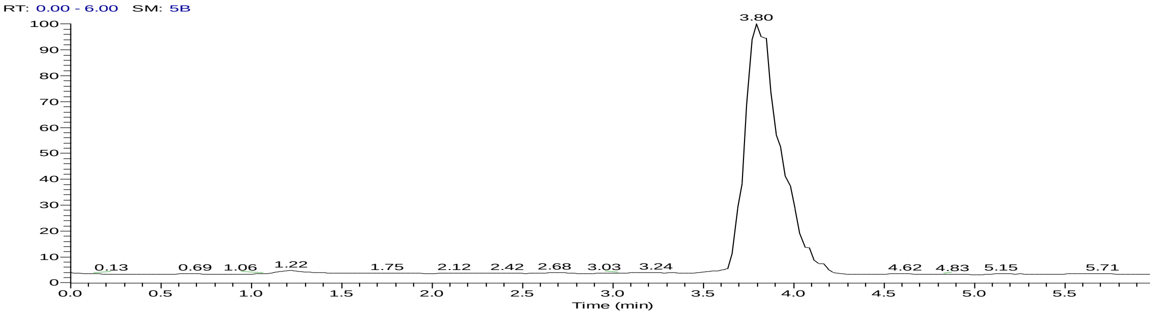

(b)

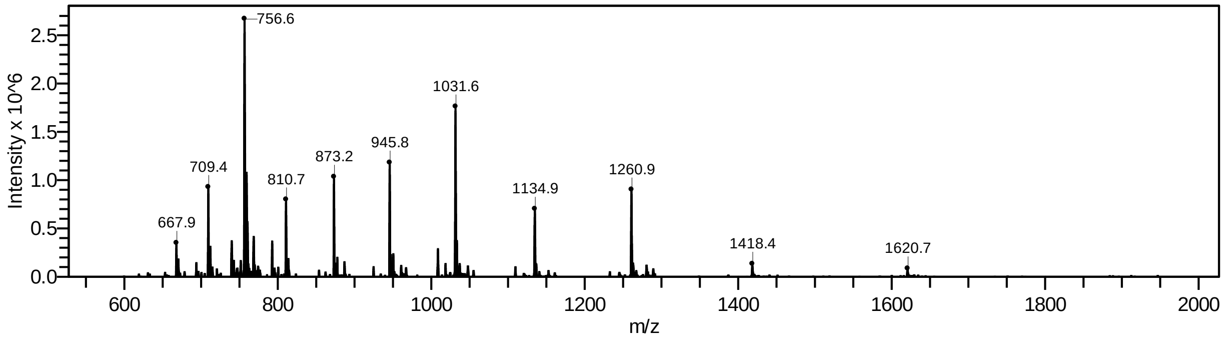

(c)

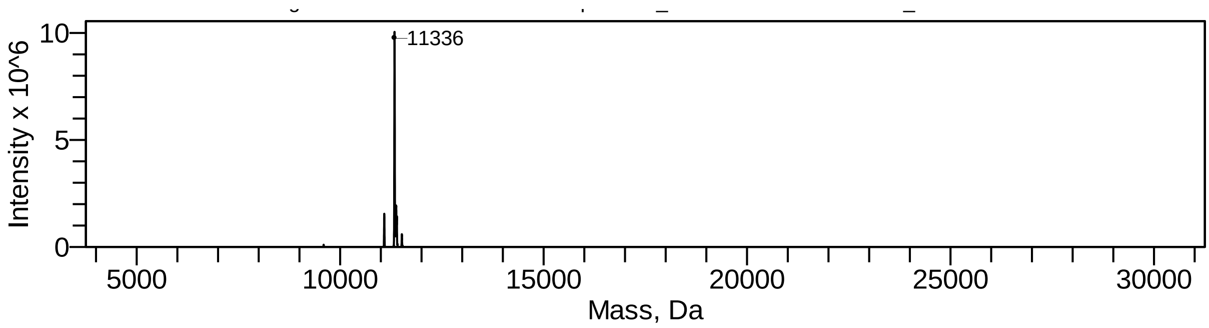

Figure S9: (a) TIC, (b) non-deconvoluted and (c) deconvoluted MS data for UbG76C-bromomaleimide.

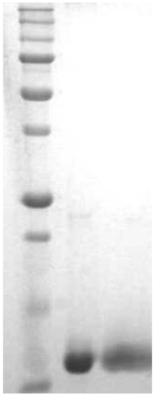

Lane: 1 2 3

Figure S10: SDS page gel UbG76C before and after dibromomaleimide conjugation. Lane: 1 – Precision Plus Protein Standards ladder (BIO-RAD); 2 – UbG76C; 3-UbG76C-bromomaleimide. 16% SDS page gel under non-reducing conditions visualised with Coomassie staining.

WT Ubiquitin with dibromomaleimide

To a solution of WT ubiquitin (Bio-Techne) (1 mg mL^{-1}, 100 μL) in either sodium phosphate buffer pH 6 (50 mM sodium phosphate, 75 mM NaCl, pH 6) or sodium phosphate buffer pH 8 (50 mM sodium phosphate, 75 mM NaCl, pH 8), dibromomaleimide (5 μL, 9 mM solution in DMF) was added. This reaction mixture was incubated at 4 °C for 24 h. Analysis using LC-MS showed no modification.

Expected mass: 8,564

Observed mass: 8,555

(a)

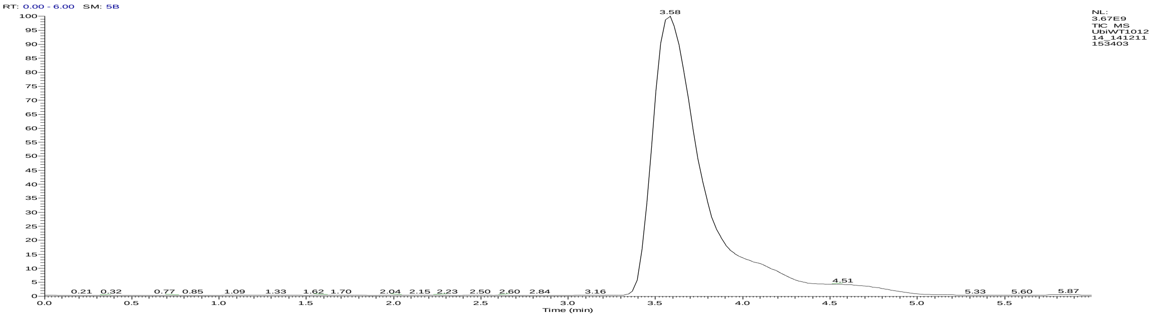

(b)

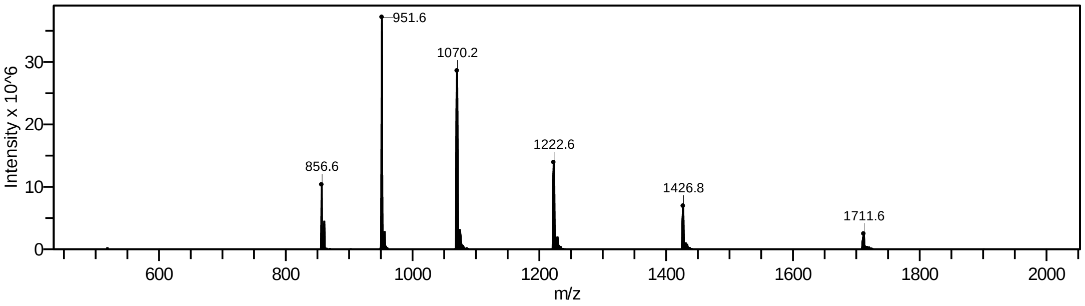

(c)

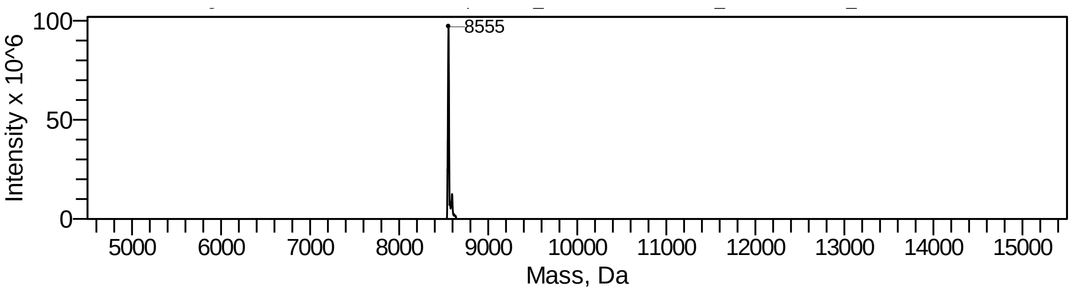

Figure S11: (a) TIC, (b) non-deconvoluted and (c) deconvoluted MS data for WT ubiquitin after incubation with dibromomaleimide.

(a)

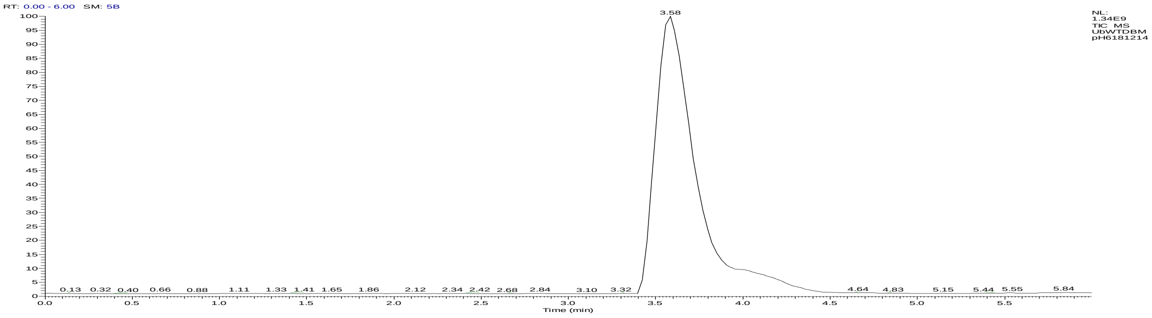

(b)

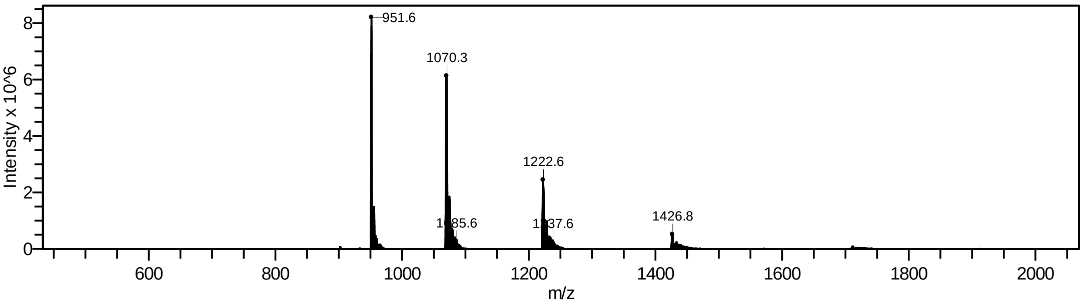

(c)

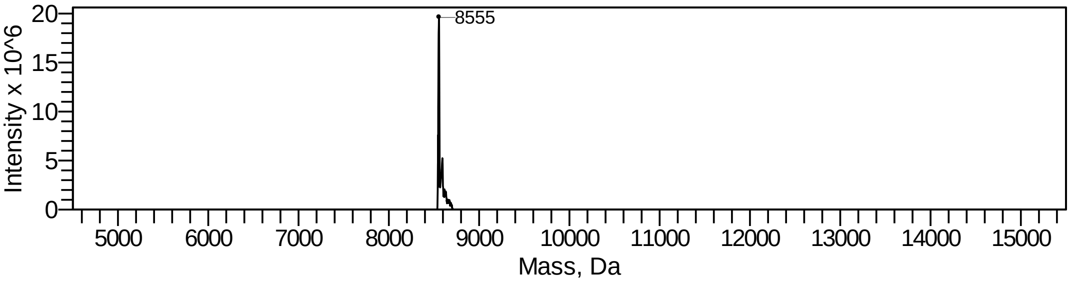

Figure S12: (a) TIC, (b) non-deconvoluted and (c) deconvoluted MS data for WT ubiquitin with dibromomaleimide at pH 6.

(a)

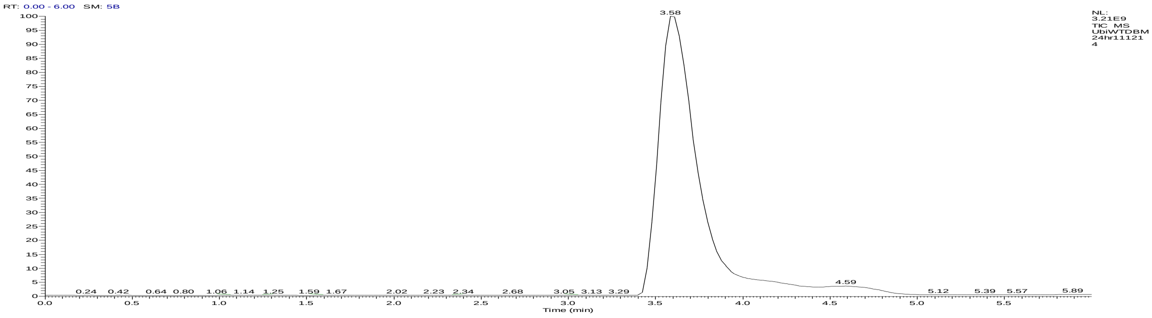

(b)

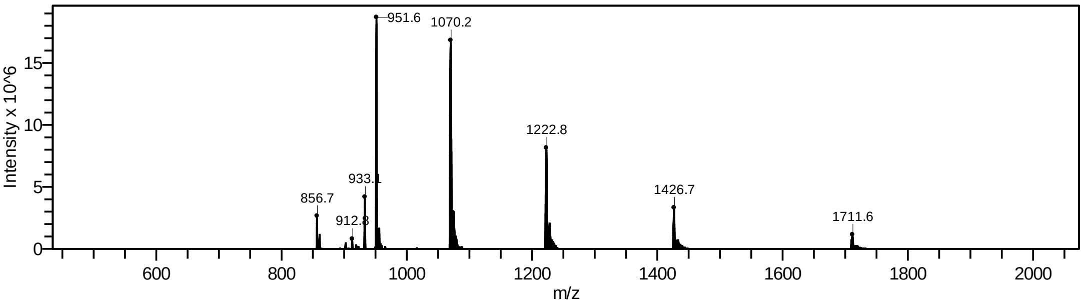

(c)

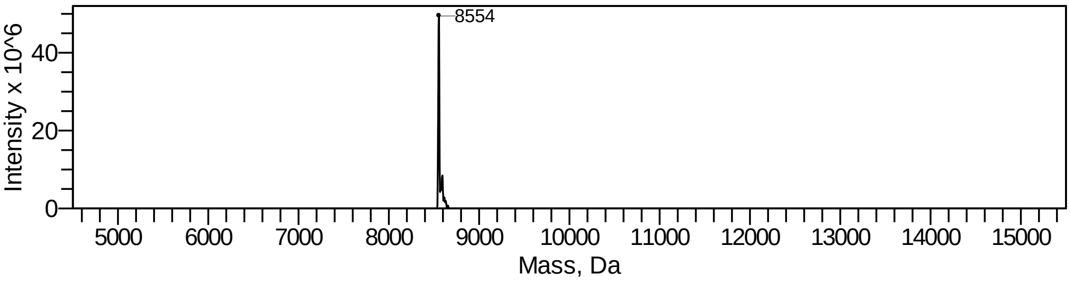

Figure S13: (a) TIC, (b) non-deconvoluted and (c) deconvoluted MS data for WT ubiquitin with dibromomaleimide at pH 8.

Ubiquitin-maleimide-Ubiquitin conjugates

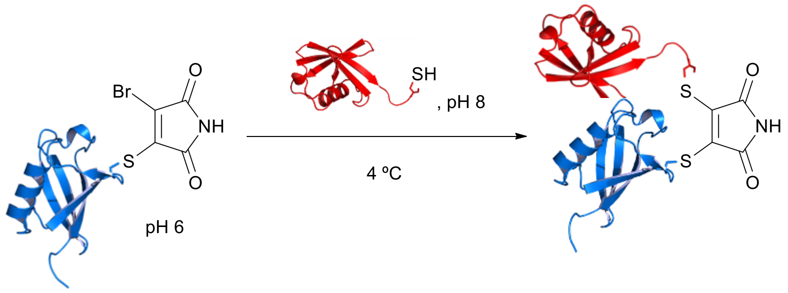

Ubiquitin-maleimide-ubiquitin conjugates were all prepared using the following method. The bromomaleimide modified ubiqutin was prepared as above, then excess dibromomaleimide was removed by ultracentrifugation using VivaSpin sample concentrators (GE Healthcare, 5,000 MWCO). Ubiquitin mutant UbG76C (1 mg mL^{-1}, 100 μL) in sodium phosphate buffer pH 6 (50 mM sodium phosphate, 75 mM NaCl, 1 mM TCEP, pH 6) was buffer exchanged by ultracentrifugation into sodium phosphate buffer pH 8 (50 mM sodium phosphate, 75 mM NaCl, pH 8). Both protein samples were concentrated to half their original volume (to give 50 μL) and mixed together in a 1:1 volume ratio, to give a final volume of 100 μL. This reaction mixture was incubated on ice for 1 h. Analysis using LC-MS showed the coupling was complete. The coupling was judged complete when the limiting species was no longer visible by LC-MS.

Due to the low extinction coefficient of A_{280} for ubiquitin (at 1 mg mL^{-1} A_{280} = 0.27) and the high A_{280} for bromomaleimide, accurate concentration measurements of the modified protein proved challenging. Therefore assumed concentrations from initial ubiquitin values were used, this resulted in the observed excess of ubiquitin or modified ubiquitin. The reaction was judged complete when only one starting material remained.

UbK27C-maleimide-UbG76C conjugate

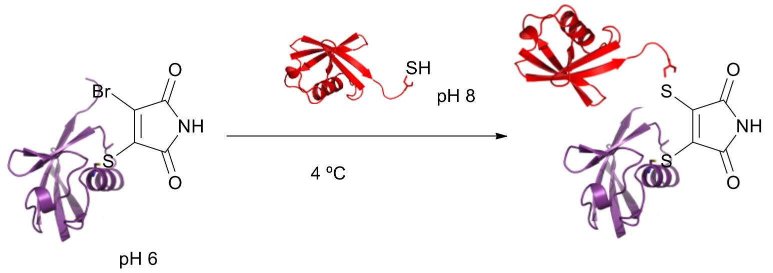

Expected mass: 22,348

Observed mass: 22,358

(a)

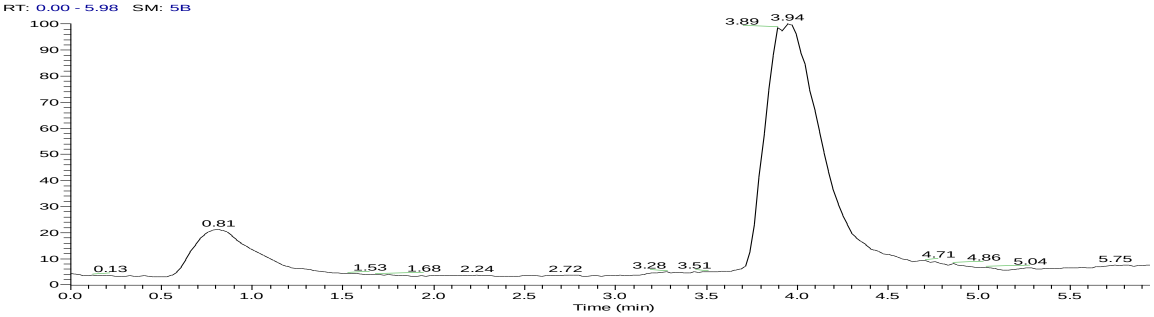

(b)

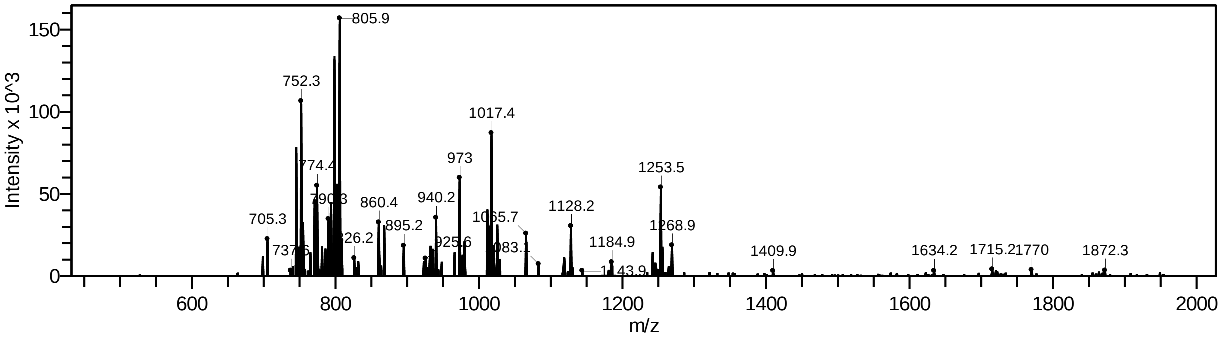

(c)

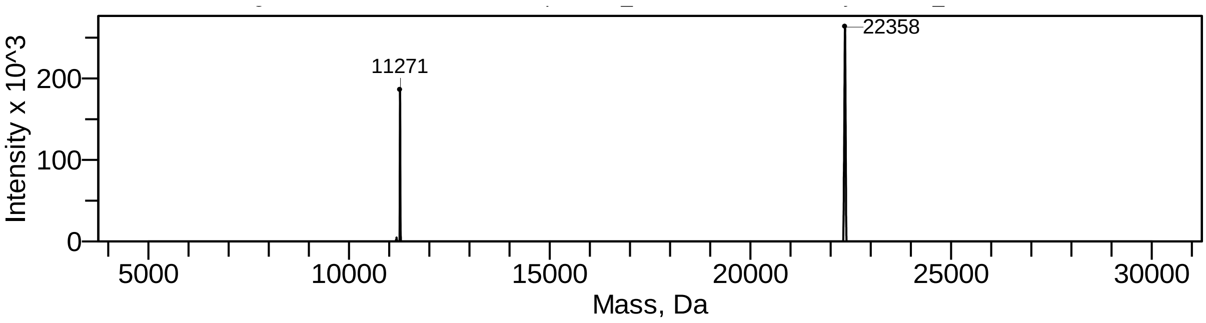

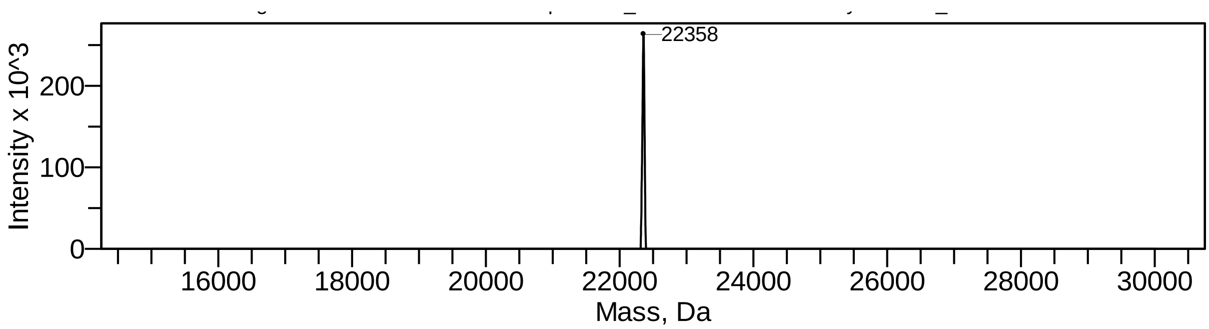

Figure S14: (a) TIC, the first (0.5-1.0 min) peak is the injection peak and does not correspond to a protein ion series, (b) non-deconvoluted and (c) deconvoluted MS data for UbK27C-maleimide-UbG76C conjugate.

UbK48C-maleimide-UbG76C conjugate

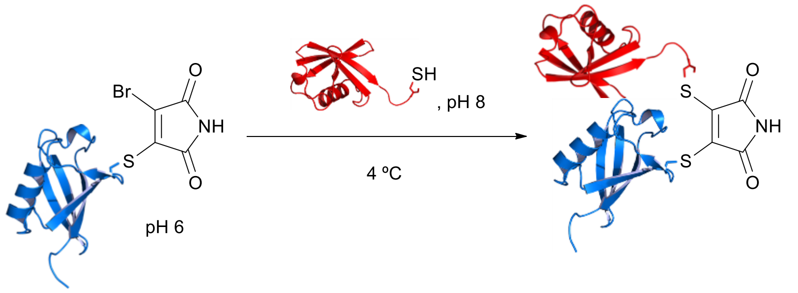

Expected mass: 22,348

Observed mass: 22,353

(a)

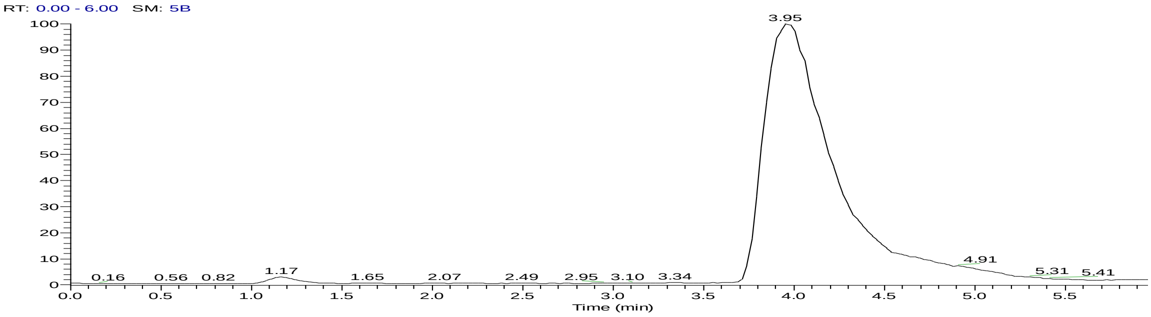

(b)

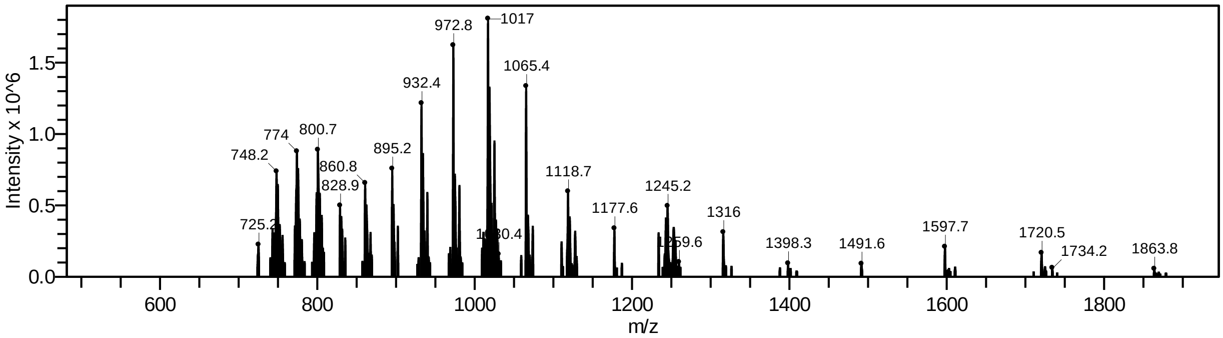

(c)

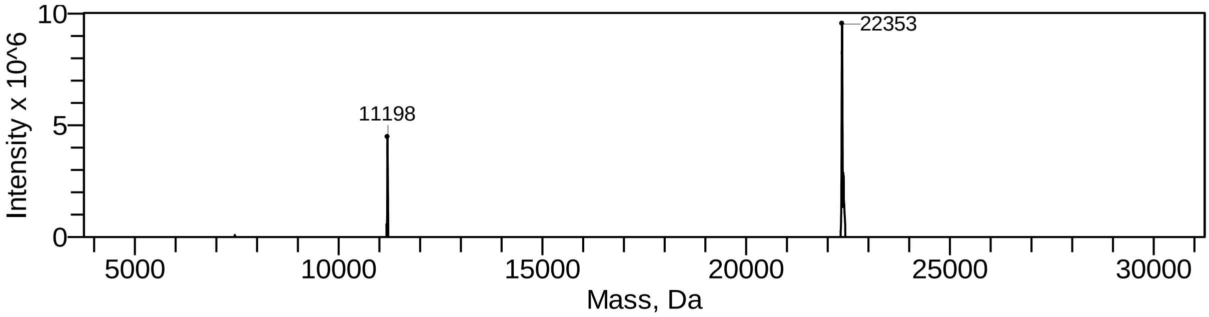

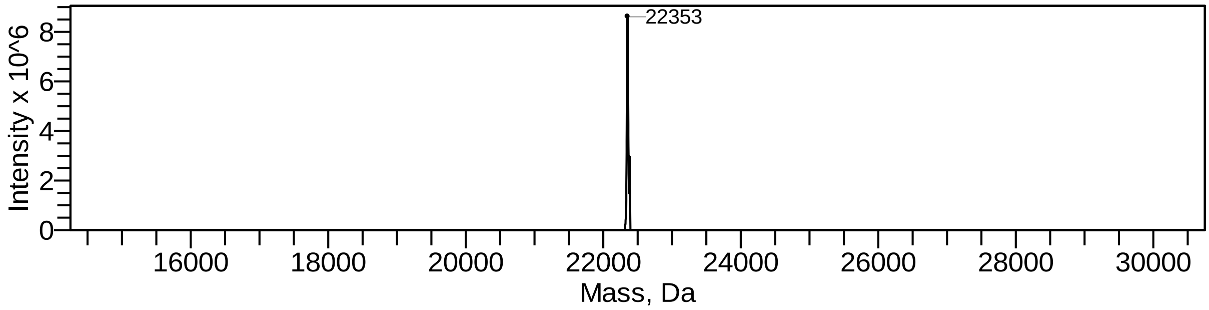

Figure S15: (a) TIC, (b) non-deconvoluted and (c) deconvoluted MS data for UbK48C-maleimide-UbG76C conjugate.

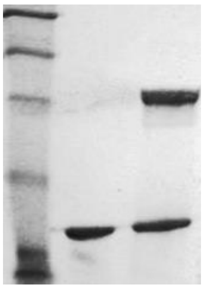

Lane: 1 2 3

Figure S16: SDS page gel for UbK48C-maleimide-UbG76C conjugation.

Lane: 1 – SeeBlue Plus2 ladder (Invitrogen); 2 – UbK48C; 3- UbK48C-maleimide-UbG76C conjugate. 15% SDS page gel under non-reducing conditions visualised with Coomassie staining.

UbK63C-maleimide-UbG76C conjugate

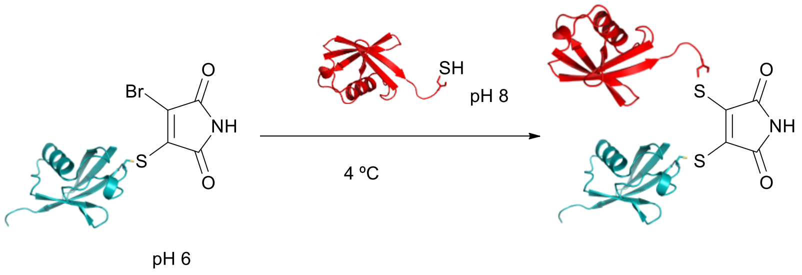

Expected mass: 22,348

Observed mass: 22,354

(a)

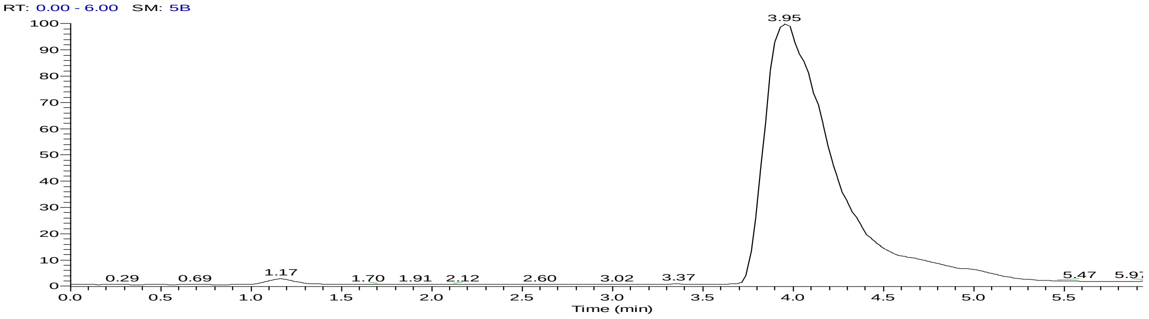

(b)

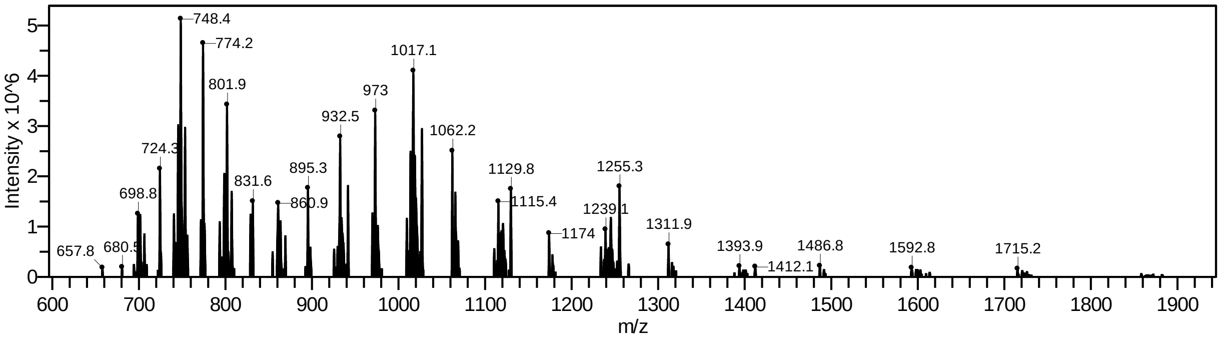

(c)

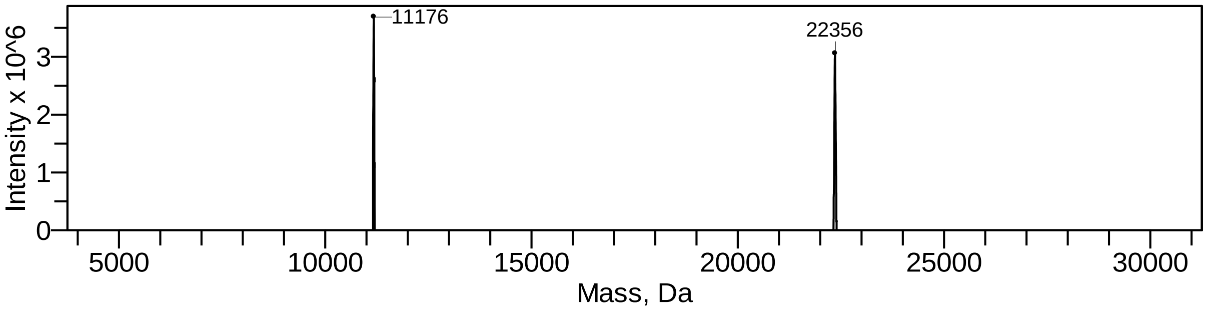

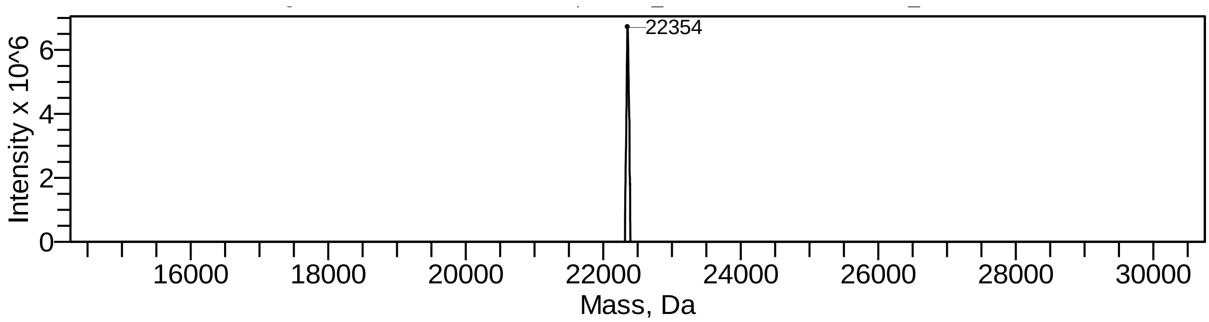

Figure S17: (a) TIC, (b) non-deconvoluted and (c) deconvoluted MS data for UbK63C-maleimide-UbG76C conjugate.

Large scale UbK48C-maleimide-UbG76C conjugate

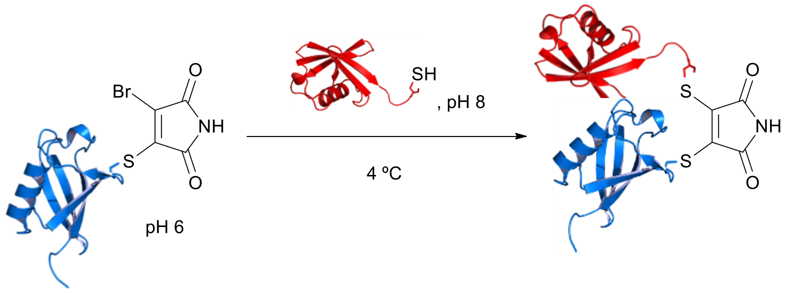

To a solution of cysteine ubiquitin mutant UbK48C (1 mg mL^{-1}, 600 µL) in sodium phosphate buffer pH 6 (50 mM sodium phosphate, 75 mM NaCl, pH 6) was added dibromomaleimide (40 µL, 9 mM solution in DMF). This reaction mixture was incubated on ice for 16 h. Analysis using LC-MS showed the complete modification of the ubiquitin. Excess dibromomaleimide was removed by ultracentrifugation using VivaSpin sample concentrators (GE Healthcare, 5,000 MWCO). Ubiquitin mutant UbG76C (1 mg mL^{-1}, 600 µL) in sodium phosphate buffer pH 6 (50 mM sodium phosphate, 75 mM NaCl, 1 mM TCEP, pH 6) was buffer exchanged by ultracentrifugation into sodium phosphate buffer pH 8 (50 mM sodium phosphate, 75 mM NaCl, pH 8). Both protein samples were concentrated to half their original volume (to give 300 µL) and mixed together in a 1:1 volume ratio, to give a final volume of 600 µL. This reaction mixture was incubated on ice for 16 h. Analysis using LC-MS showed the coupling was complete. The conjugate was loaded onto a Superdex 75 16/60 size exclusion column (GE Healthcare) equilibrated with 50 mM sodium phosphate buffer, pH 7, 75 mM NaCl. Two peaks were eluted off the column, with masses of ~25 kDa and ~13 kDa. The fractions for each peak were pooled and concentrated. Analysis by mass spectrometry confirmed that the first peak contained the UbK48C-maleimide-UbG76C conjugate. A solution of 470 µL (at the same concentration as the original cysteine ubiquitin mutant UbK48C) of the first peak was obtained, thus giving a yield of 78% for the UbK48C-maleimide-UbG76C conjugate.

Expected mass: 22,348

Observed mass: 22,352

Reaction mixture:

(a)

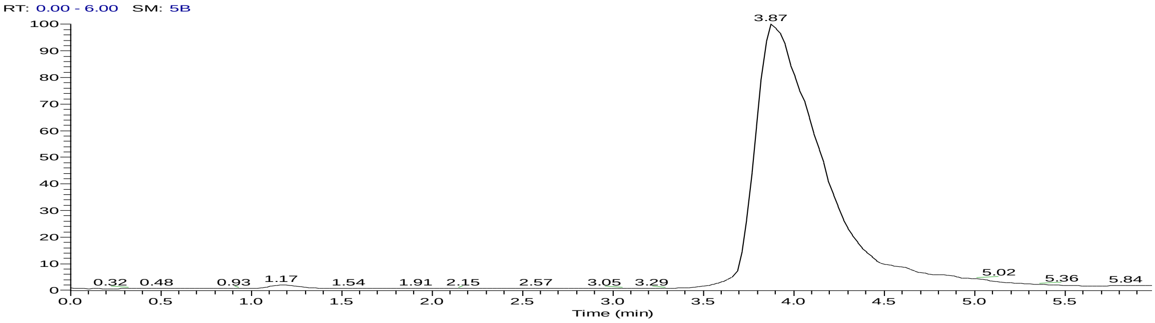

(b)

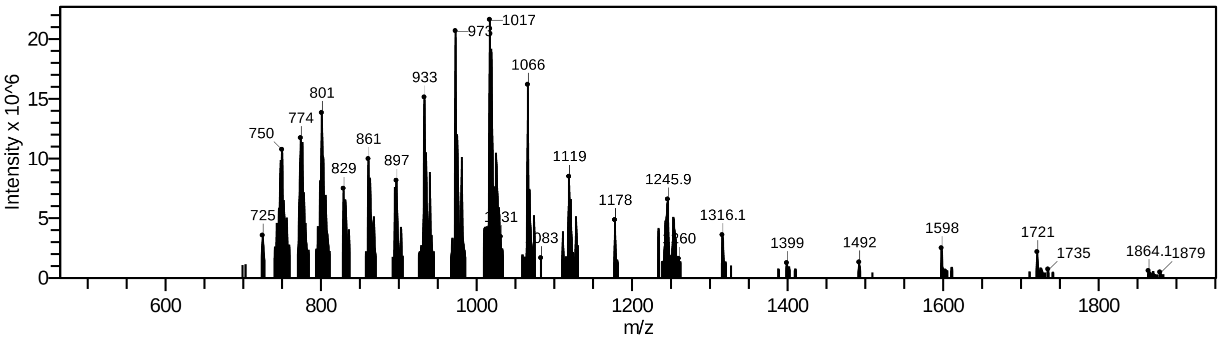

(c)

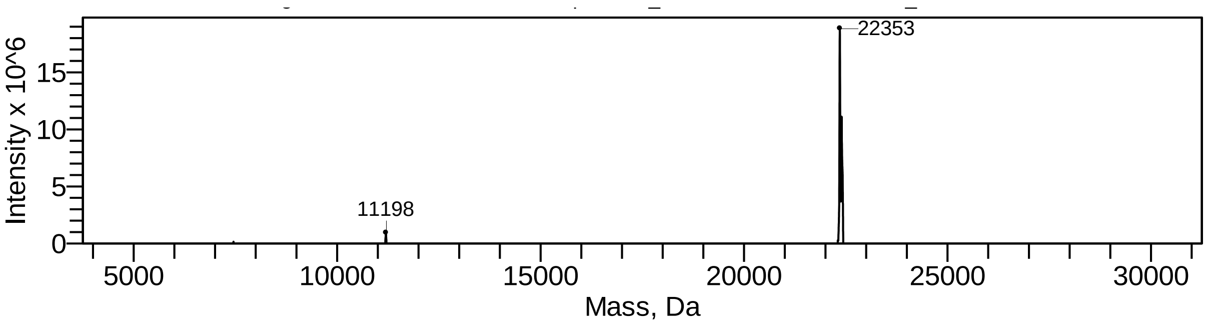

Figure S18: (a) TIC, (b) non-deconvoluted and (c) deconvoluted MS data for UbK48C-maleimide-UbG76C conjugate large scale reaction mixture.

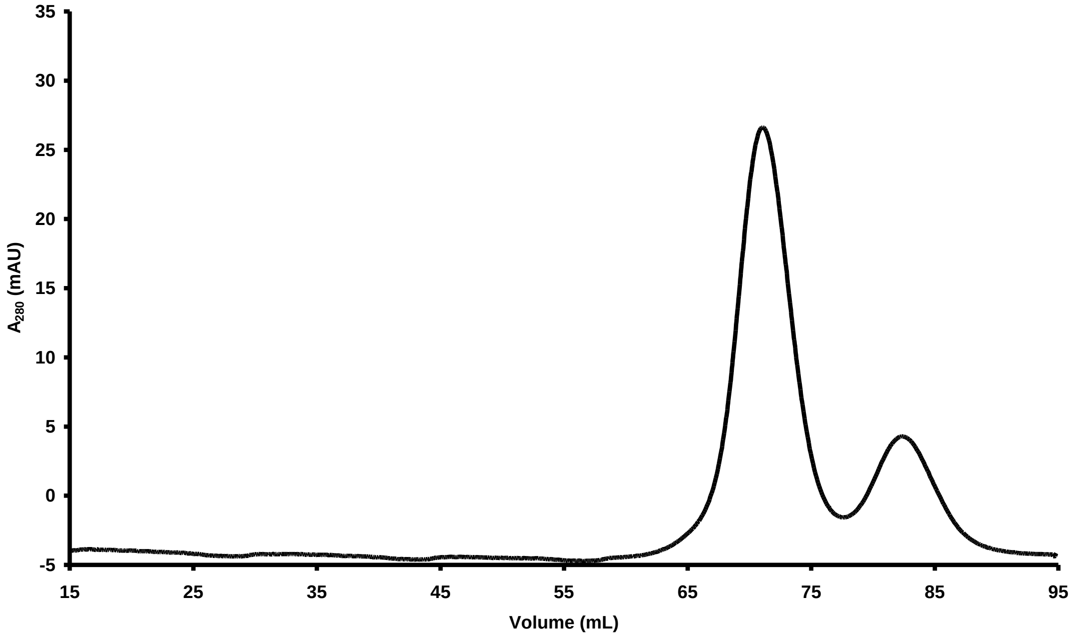

Figure S19: Size exclusion chromatogram of UbK48C-maleimide-UbG76C conjugate large scale reaction mixture.

Peak 1, UbK48C-maleimide-UbG76C conjugate:

(a)

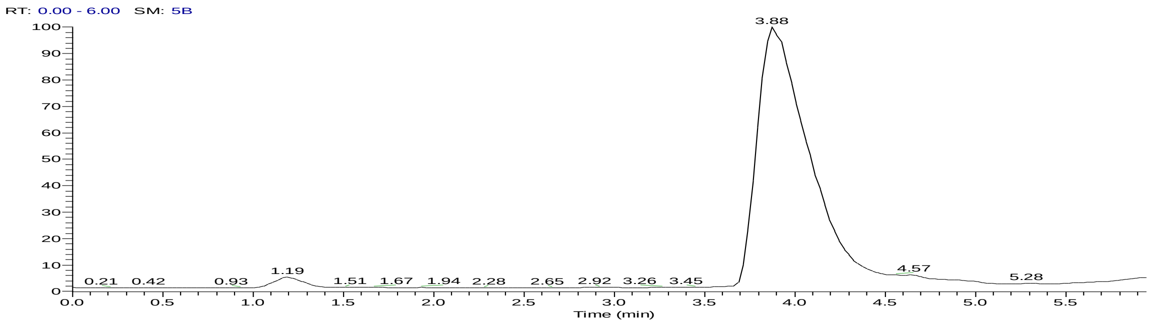

(b)

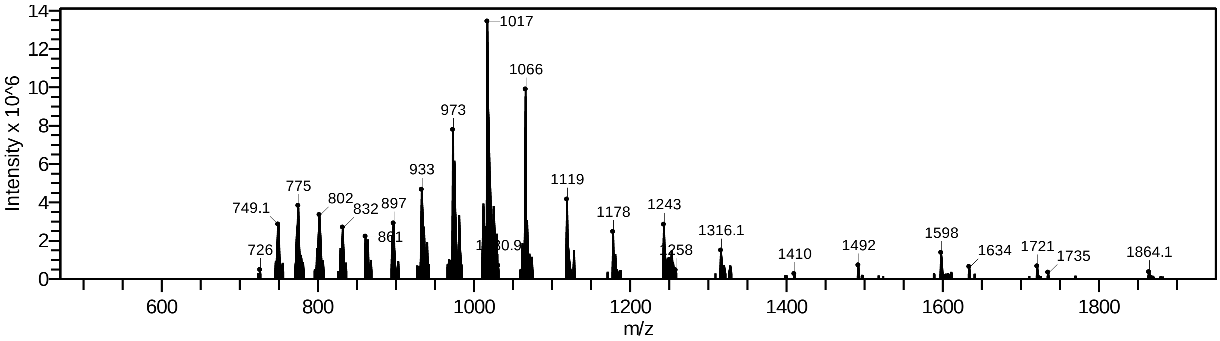

(c)

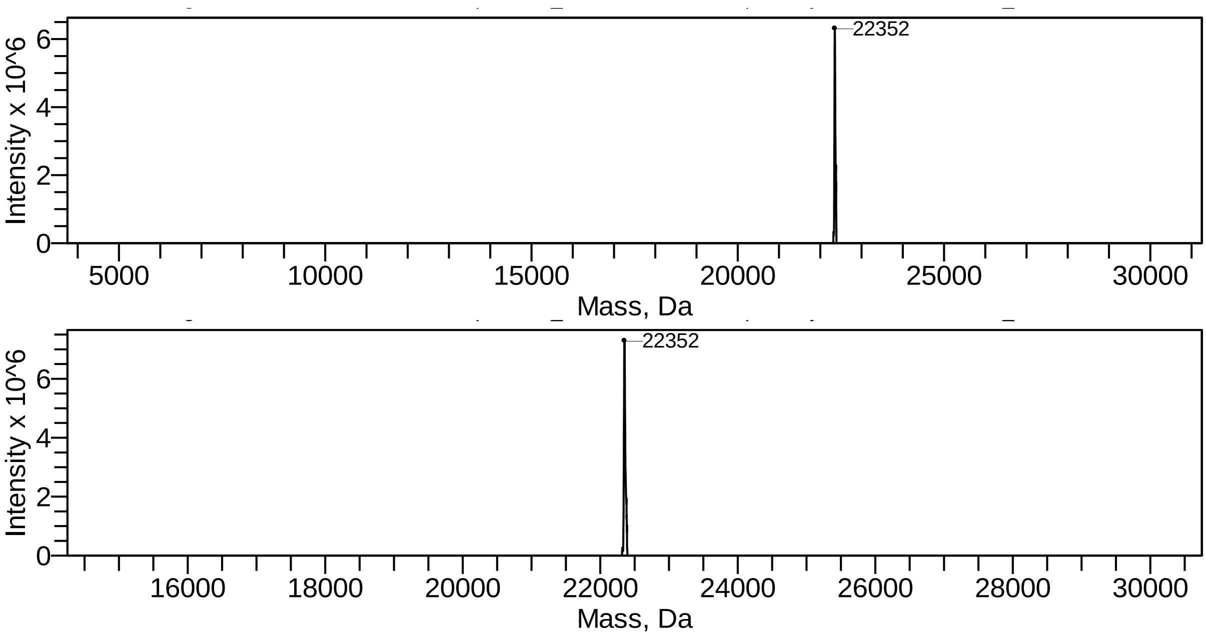

Figure S20: (a) TIC, (b) non-deconvoluted and (c) deconvoluted MS data for peak one from the size exclusion purified UbK48C-maleimide-UbG76C conjugate reaction mixture.

Ubiqutin-maleimide-GFP conjugates

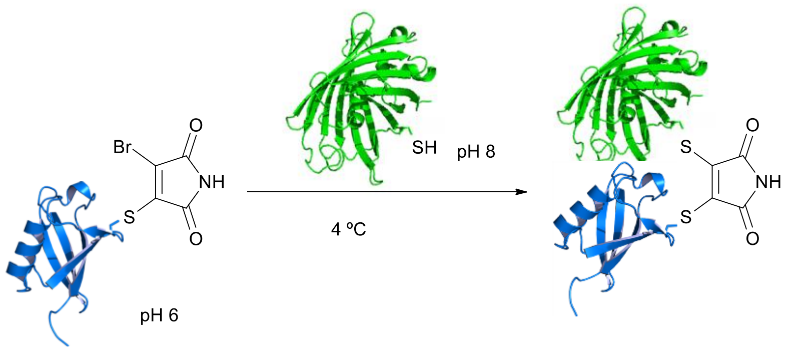

Ubiquitin-maleimide-GFP conjugates were prepared using the following method. The bromomaleimide modified ubiqutin was prepared as previously stated, then excess dibromomaleimide was removed by ultracentrifugation using VivaSpin sample concentrators (GE Healthcare, 5,000 MWCO). GFP mutant S147C (2.6 mg mL^{-1}, 100 μL) in PBS was buffer exchanged by ultracentrifugation into sodium phosphate buffer pH 8 (50 mM sodium phosphate, 75 mM NaCl, pH 8). Both protein samples were concentrated to half their original volume (to give 50 μL) and mixed together in a 1:1 volume ratio, to give a final volume of 100 μL. This reaction mixture was incubated on ice for 1 h. Analysis using LC-MS showed the coupling was complete.

UbK48C-maleimide-GFPS147C conjugate

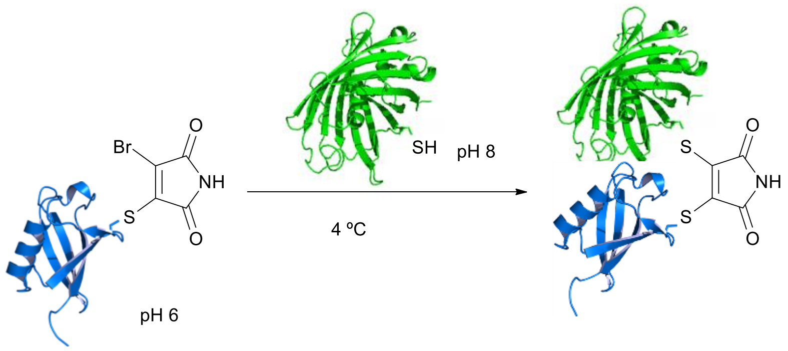

Expected mass: 40,529

Observed mass: 40,533

(a)

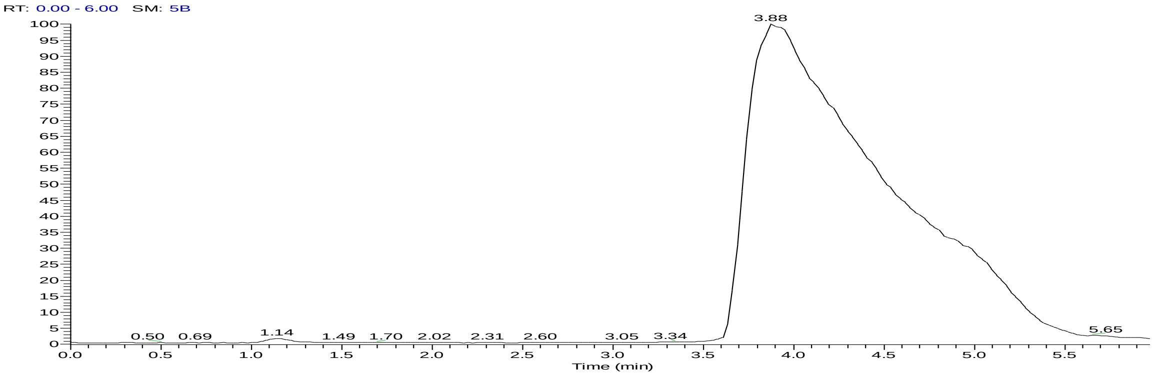

(b)

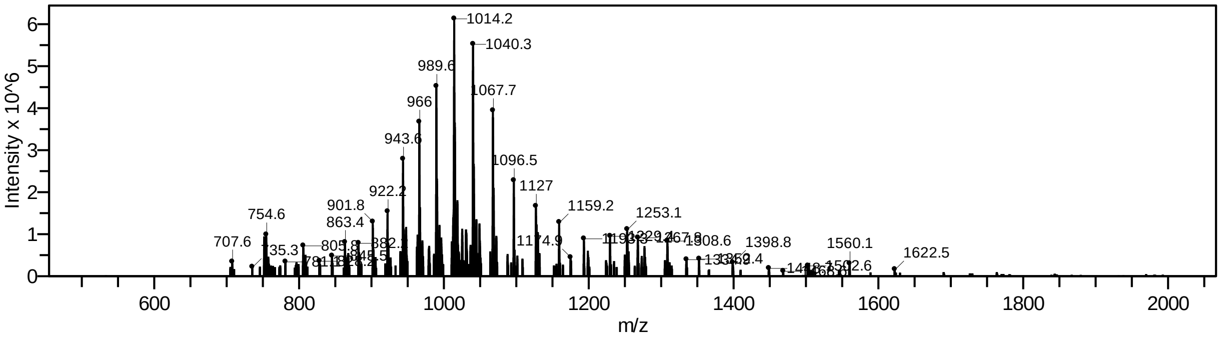

(c)

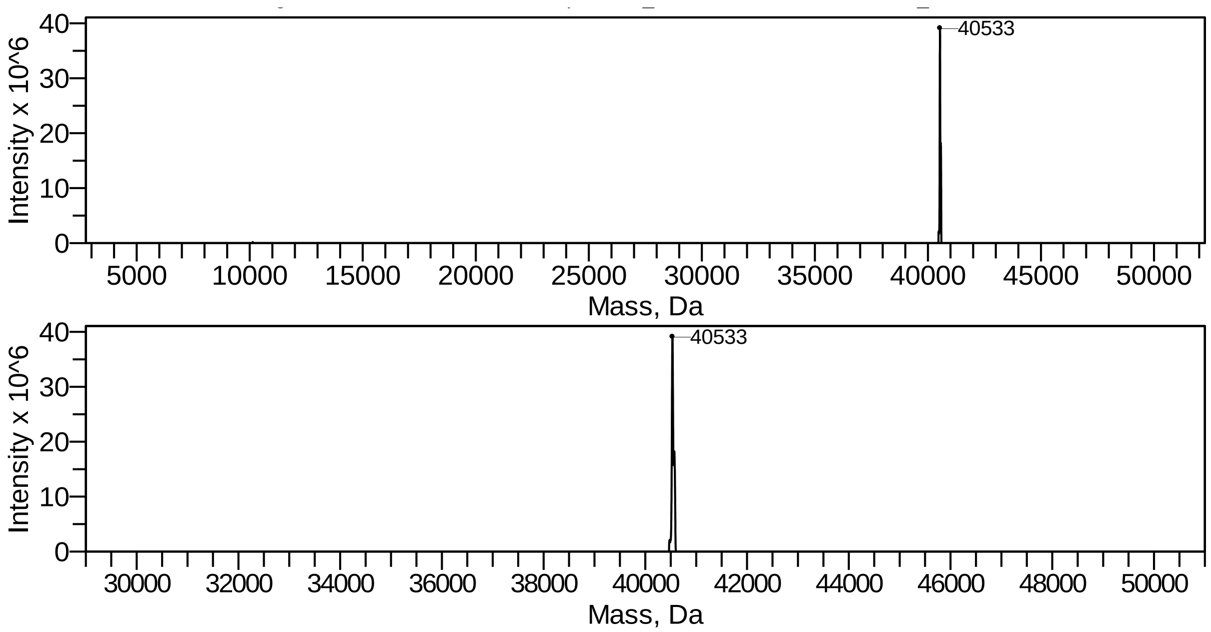

Figure S21: (a) TIC, (b) non-deconvoluted and (c) deconvoluted MS data for UbK48C-maleimide-GFPS147C conjugate.

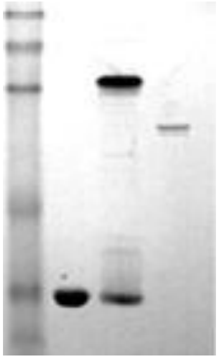

Lane: 1 2 3 4

Figure S22: SDS page gel OF UbK48C-maleimide-GFPS147C conjugation.

Lane: 1 – Seeblue Plus2 ladder (Invitrogen); 2 – UbK48C; 3- UbK48C-maleimide-GFPS147C conjugate, 4-GFPS147C. 16% SDS page gel under non-reducing conditions visualised with Coomassie staining.

UbG76C-maleimide-GFPS147C conjugate

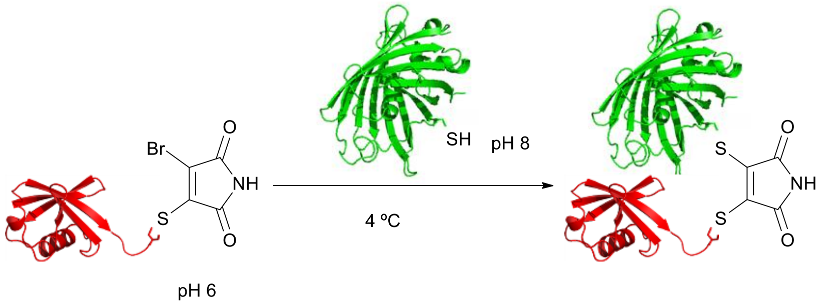

Expected mass: 40,600

Observed mass: 40,603

(a)

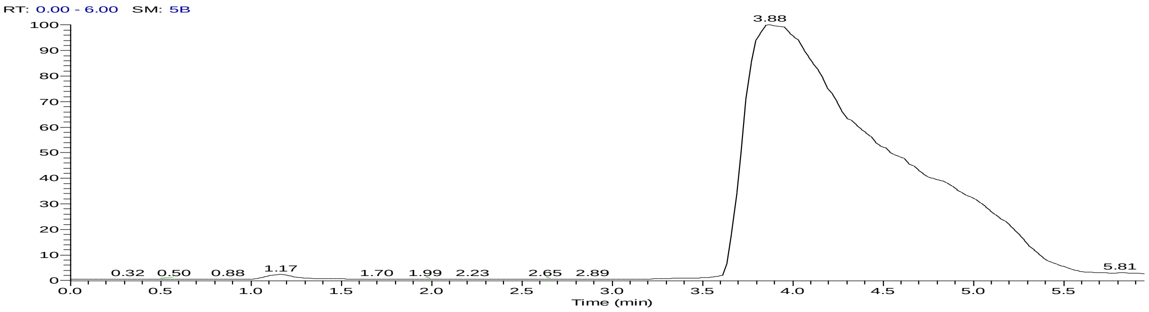

(b)

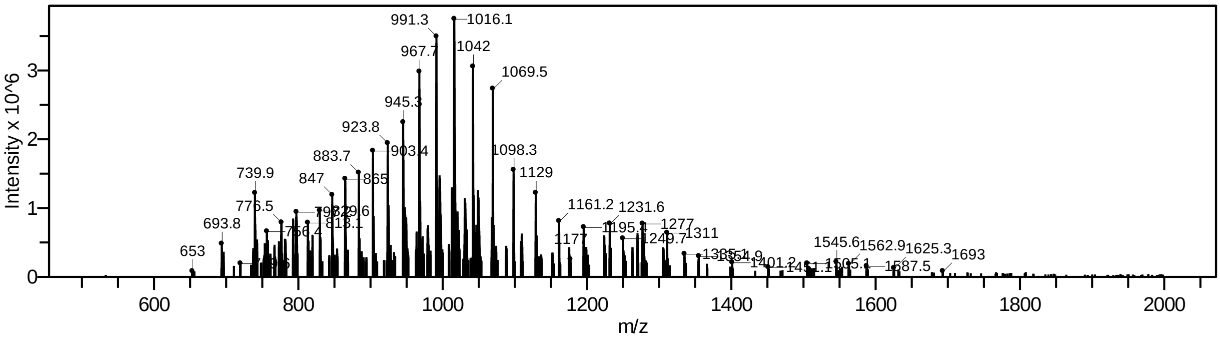

(c)

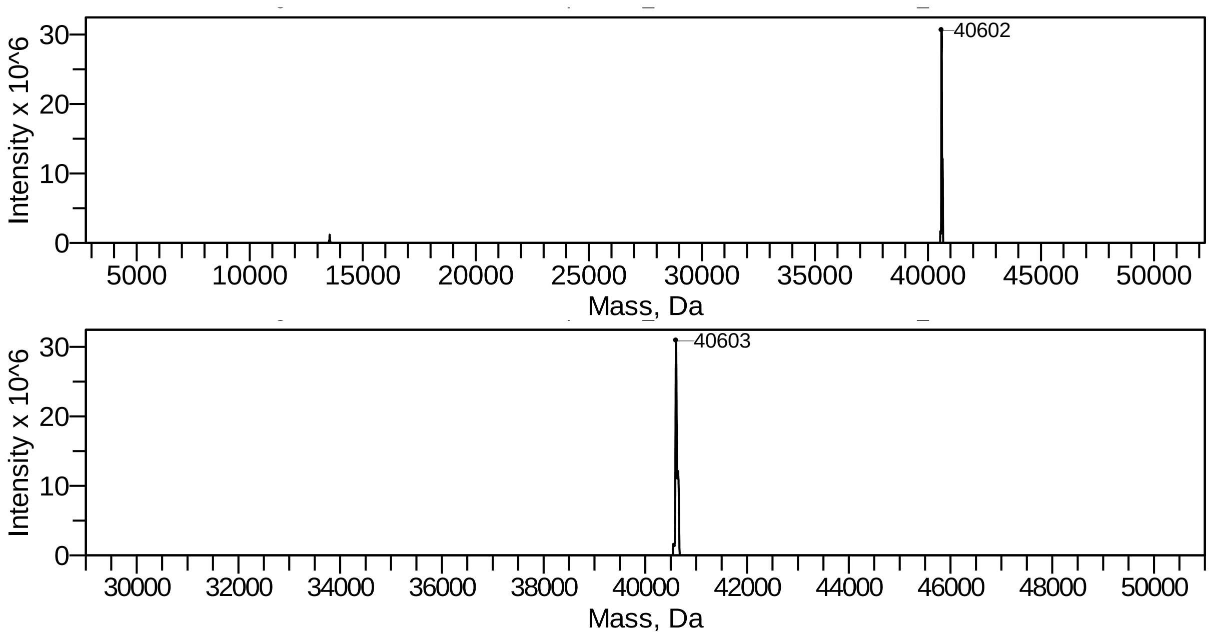

Figure S23: (a) TIC, (b) non-deconvoluted and (c) deconvoluted MS data for UbG76C-maleimide-GFPS147C conjugate.

Pyridazinedione modified ubiquitin

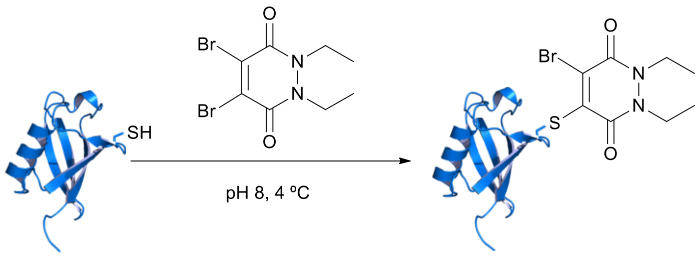

Modification of all cysteine ubiquitin mutants with dibromopyridazinedione was achieved using the following method. The cysteine ubiquitin mutant UbXXC was buffer exchanged by ultracentrifugation into sodium phosphate buffer pH 8 (50 mM sodium phosphate, 75 mM NaCl, pH 8). Dibromopyridazinedione (5 μL, 90 mM solution in DMF) was added to the ubiquitin mutant UbXXC (1 mg mL^{-1}, 100 μL). This reaction mixture was incubated on ice for 1 h. Analysis using LC-MS showed complete modification of the cysteine ubiquitin mutant.

UbK27C-bromopyridazinedione

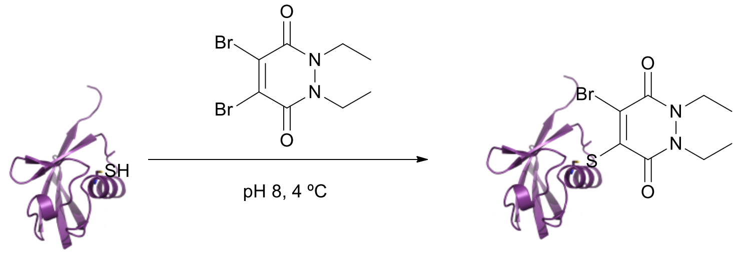

Expected mass: 11,336

Observed mass: 11,340

(a)

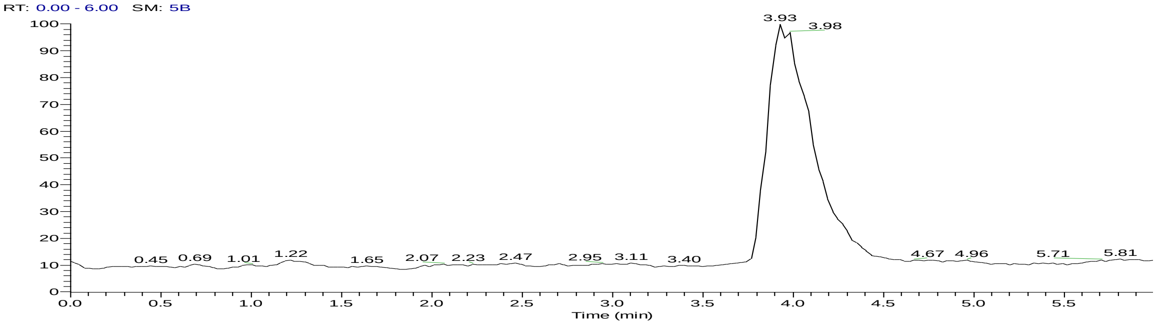

(b)

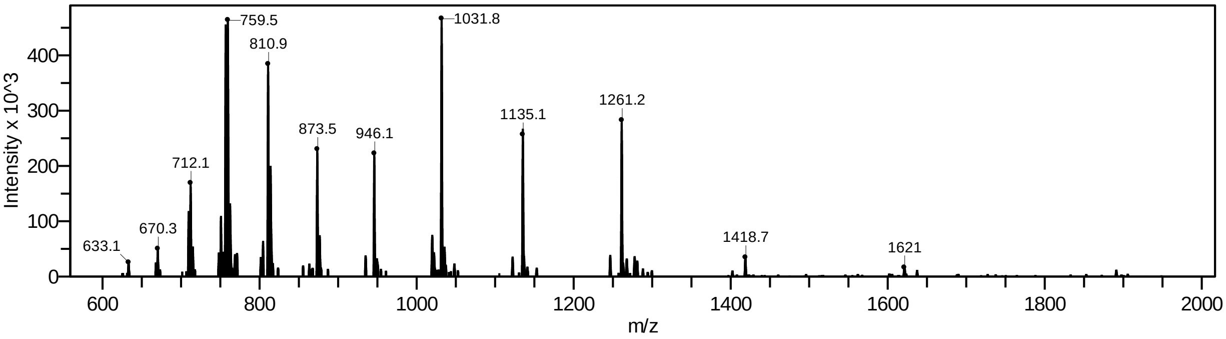

(c)

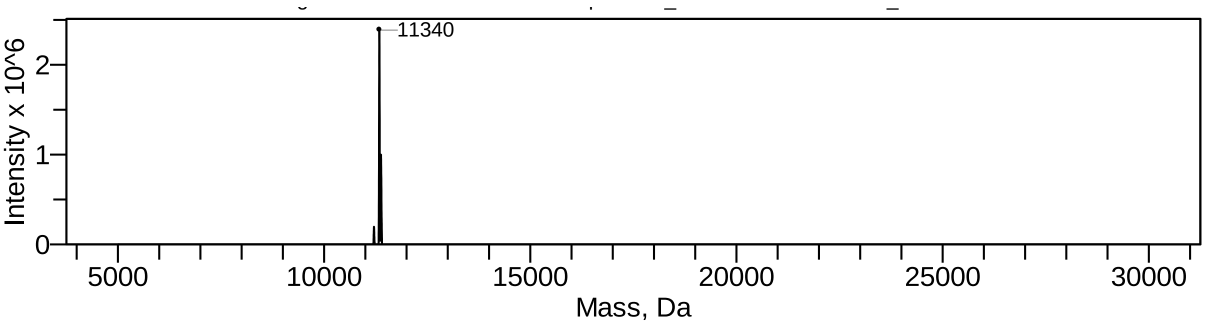

Figure S24: (a) TIC, (b) non-deconvoluted and (c) deconvoluted MS data for UbK27C-bromopyridazinedione.

UbK48C-bromopyridazinedione

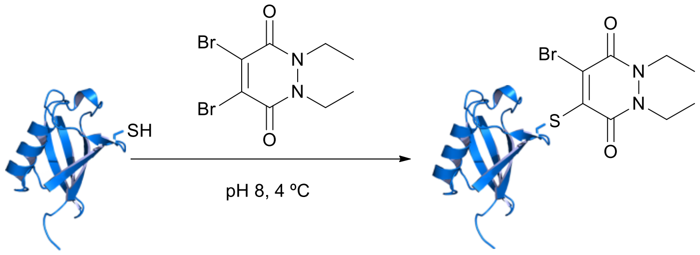

Expected mass: 11,336

Observed mass: 11,342

(a)

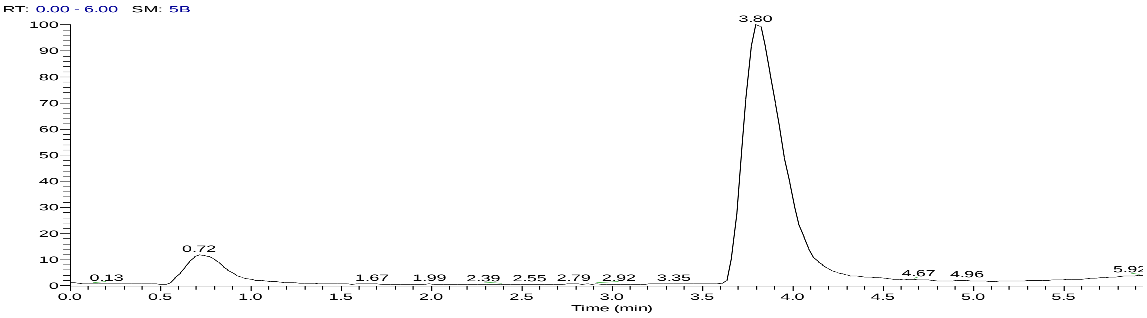

(b)

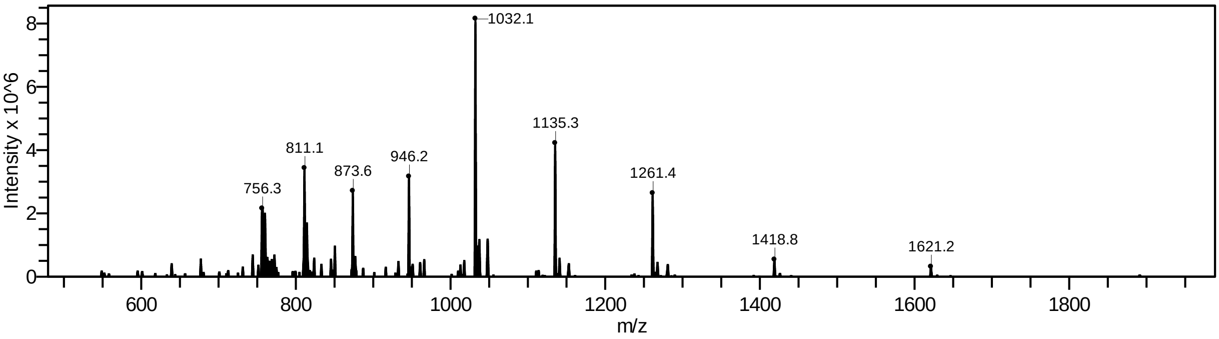

(c)

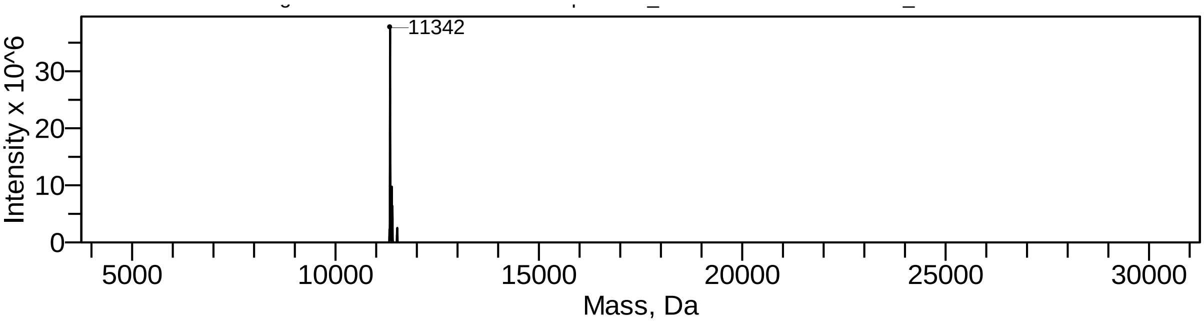

Figure S25: (a) TIC, the first (0.5-1.0 min) peak is the injection peak and does not correspond to a protein ion series, (b) non-deconvoluted and (c) deconvoluted MS data for UbK48C-bromopyridazinedione.

UbK63C-bromopyridazinedione

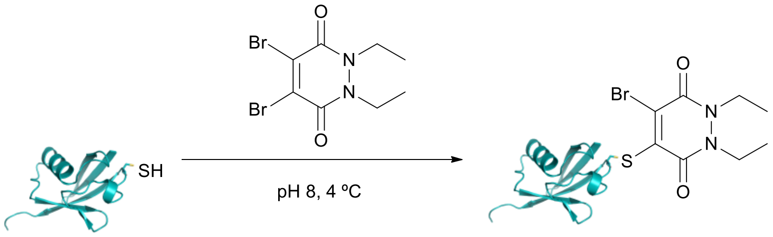

Expected mass: 11,336

Observed mass: 11,336

(a)

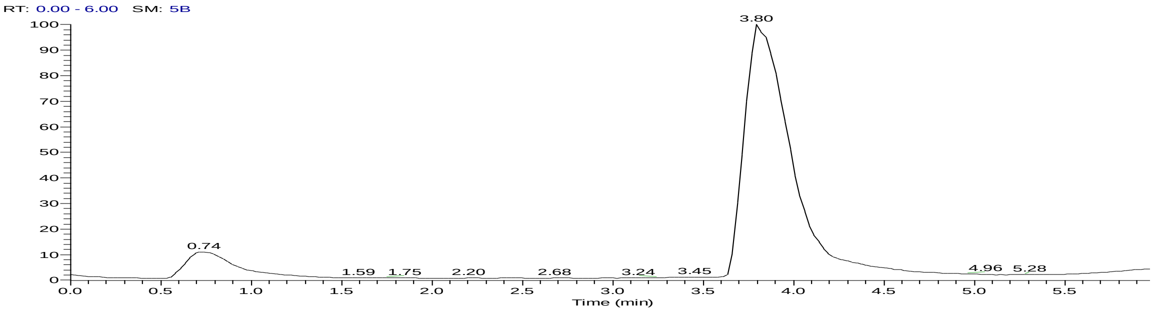

(b)

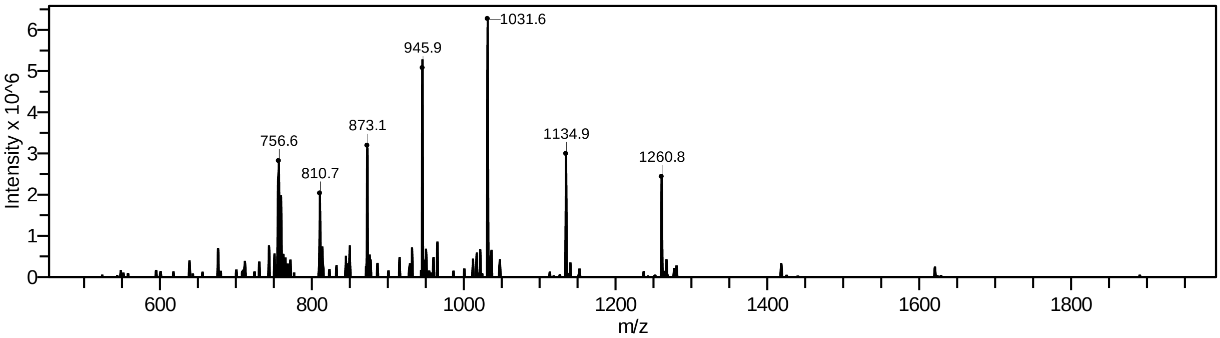

(c)

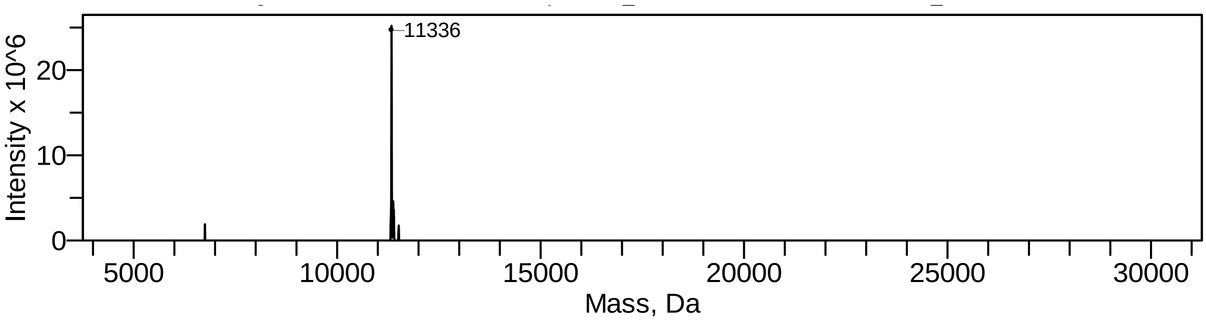

Figure S26: (a) TIC, the first (0.5-1.0 min) peak is the injection peak and does not correspond to a protein ion series, (b) non-deconvoluted and (c) deconvoluted MS data for UbK63C-bromopyridazinedione.

UbG76C-bromopyridazinedione

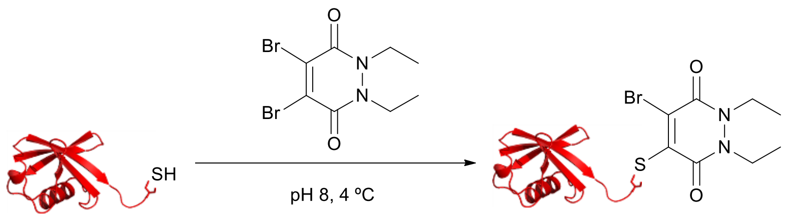

Expected mass: 11,407

Observed mass: 11,406

(a)

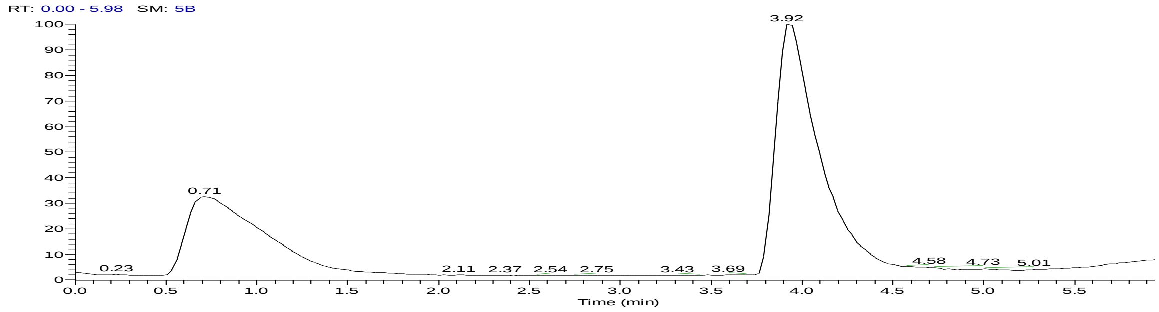

(b)

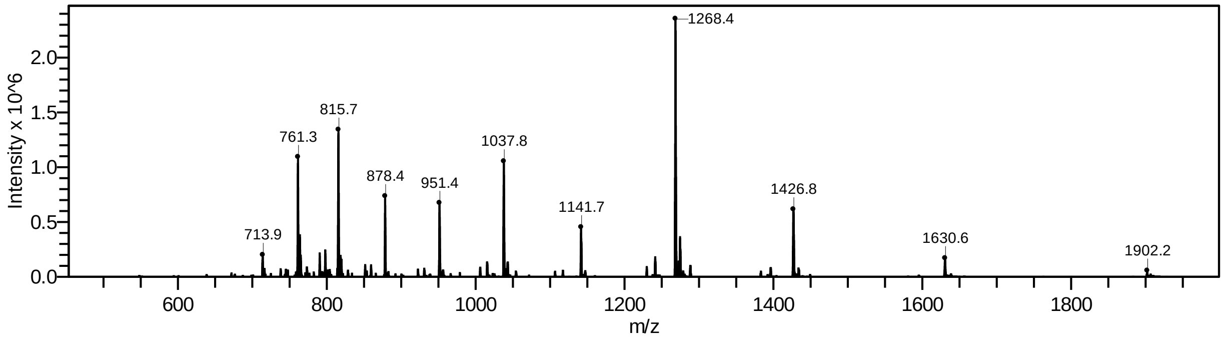

(c)

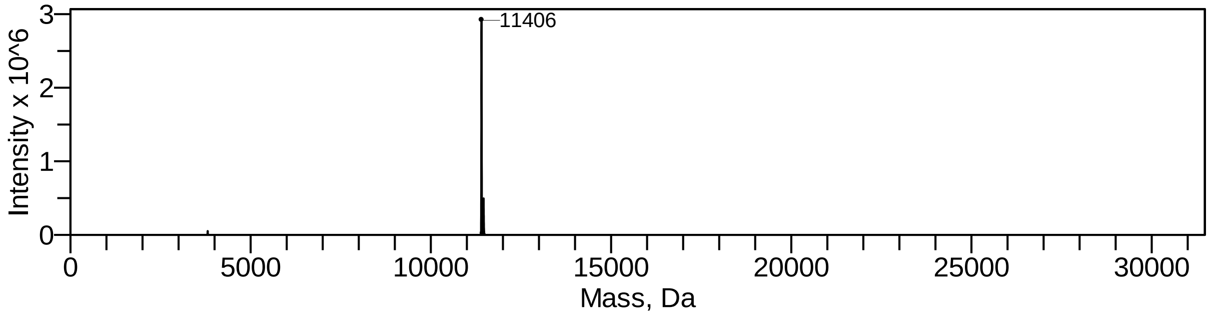

Figure S27: (a) TIC, the first (0.5-1.5 min) peak is the injection peak and does not correspond to a protein ion series, (b) non-deconvoluted and (c) deconvoluted MS data for UbG76C-bromopyridazinedione.

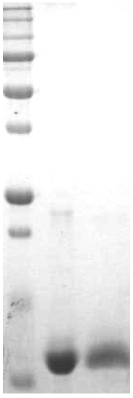

Lane: 1 2 3

Figure S28: SDS page gel for UbG76C before and after pyridazinedione conjugation. Lane: 1 – Precision Plus Protein Standards ladder (BIO-RAD); 2 – UbG76C; 3- UbG76C-bromopyridazinedione. 16% SDS page gel under non-reducing conditions visualised with Coomassie staining.

WT Ubiquitin with dibromopyridazinedione

To a solution of WT ubiquitin (Bio-Techne) (1 mg mL^{-1}, 100 μL) in sodium phosphate buffer pH 8 (50 mM sodium phosphate, 75 mM NaCl, pH 8), dibromopyridazinedione (5 μL, 90 mM solution in DMF) was added. This reaction mixture was incubated at 4 °C for 24 h. Analysis using LC-MS showed no modification.

Expected mass: 8,564

Observed mass: 8,555

(a)

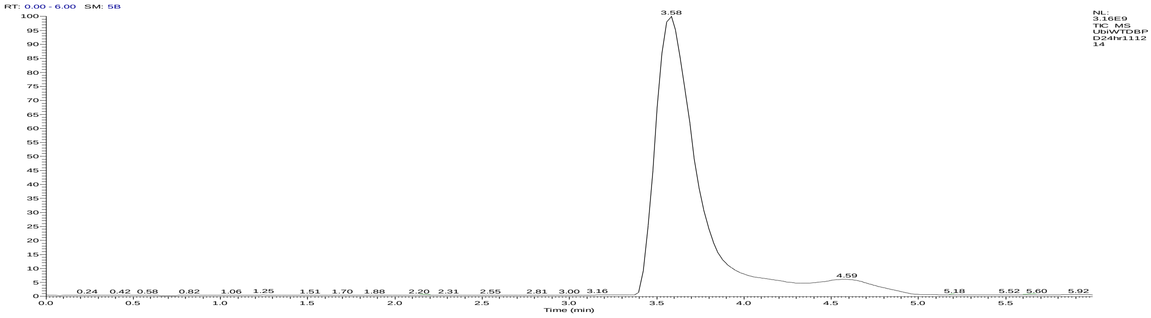

(b)

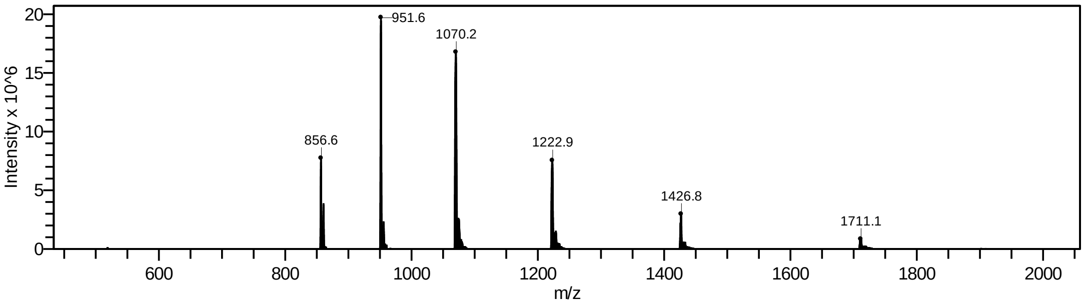

(c)

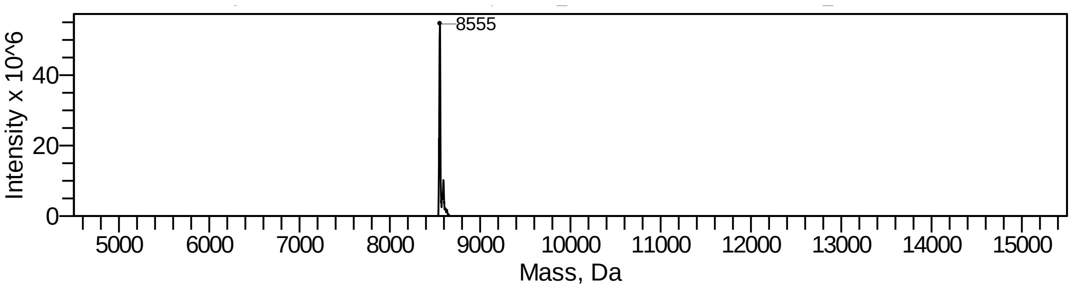

Figure S29: (a) TIC, (b) non-deconvoluted and (c) deconvoluted MS data for WT ubiquitin after incubation with dibromopyridazinedione.

Ubiquitin-pyridazinedione-Ubiquitin conjugates

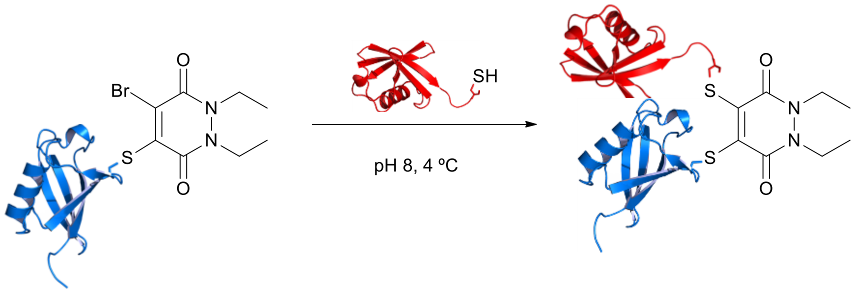

Ubiquitin-pyridazinedione-ubiquitin conjugates were prepared using the following method. The bromopyridazinedione modified ubiqutin was prepared as above, then excess dibromopyridazinedione was removed by ultracentrifugation using VivaSpin sample concentrators (GE Healthcare, 5,000 MWCO). Ubiquitin mutant UbG76C (1 mg mL^{-1}, 100 μL) in sodium phosphate buffer pH 6 (50 mM sodium phosphate, 75 mM NaCl, 1 mM TCEP, pH 6) was buffer exchanged by ultracentrifugation into sodium phosphate buffer pH 8 (50 mM sodium phosphate, 75 mM NaCl, pH 8). Both protein samples were concentrated to half their original volume (to give 50 μL) and mixed together in a 1:1 volume ratio, to give a final volume of 100 μL. This reaction mixture was incubated on ice for 16 h. Analysis using LC-MS showed the coupling was complete.

The high A_{280} for dibromopyridazinedione resulted in the same difficulties as for bromomaleimide with regards accurate concentration measurements of the modified protein. Therefore, as before, assumed concentrations from initial ubiquitin values were used, this resulted in the observed excess of ubiquitin or modified ubiquitin. The reaction was judged complete when only one starting material remained.

UbK48C-pyridazinedione-UbG76C conjugate

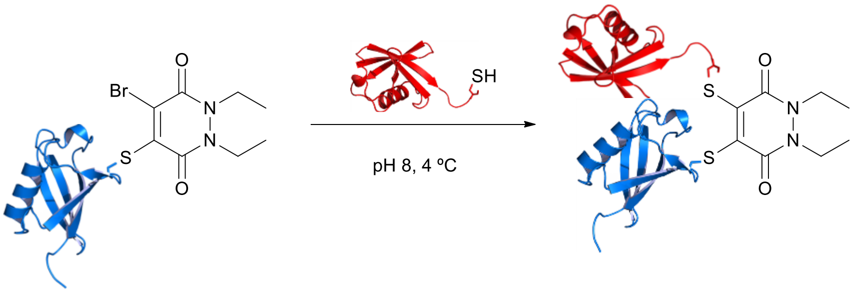

Expected mass: 22,419

Observed mass: 22,426

(a)

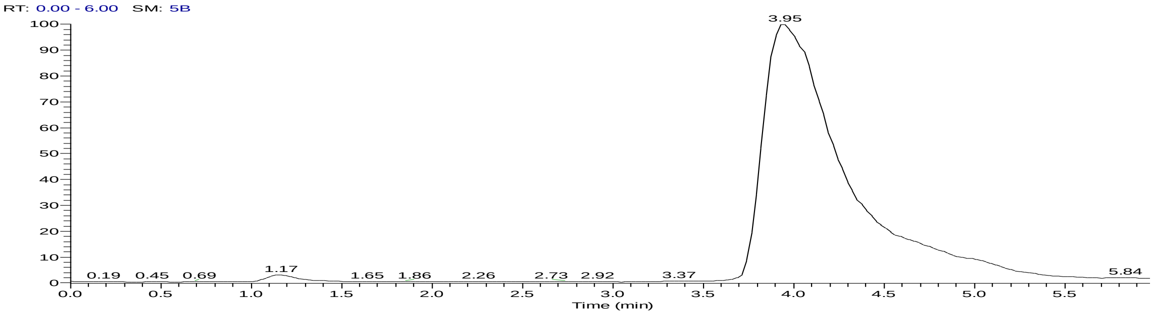

(b)

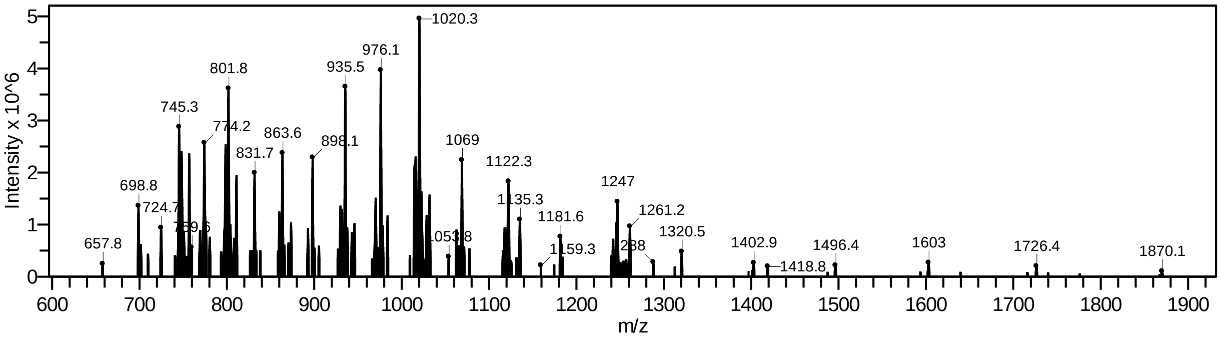

(c)

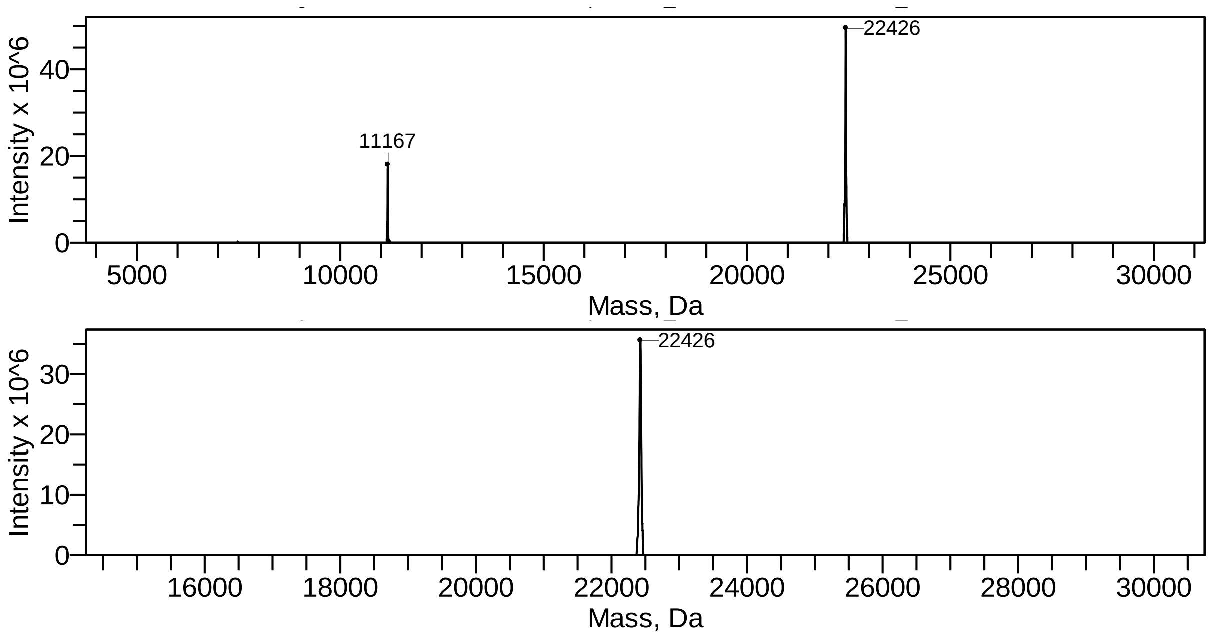

Figure S30: (a) TIC, (b) non-deconvoluted and (c) deconvoluted MS data for UbK48C-pyridazinedione-UbG76C conjugate.

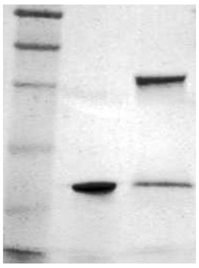

Lane: 1 2 3

Figure S31: SDS page gel UbK48C-pyridazinedione-UbG76C conjugation.

Lane: 1 – Seeblue Plus2 ladder (Invitrogen); 2 – UbK48C; 3 – UbK48C-pyridazinedione-UbG76C conjugate. 15% SDS page gel under non-reducing conditions visualised with Coomassie staining.

UbK63C-pyridazinedione-UbG76C conjugate

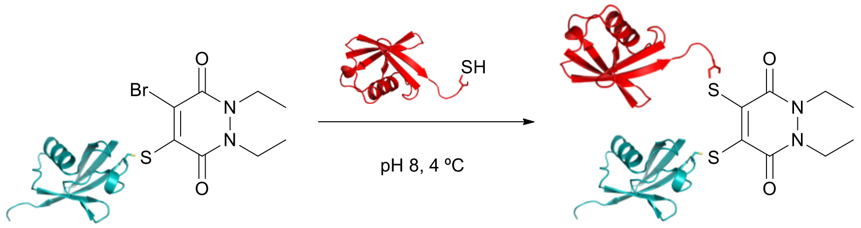

Expected mass: 22,419

Observed mass: 22,425

(a)

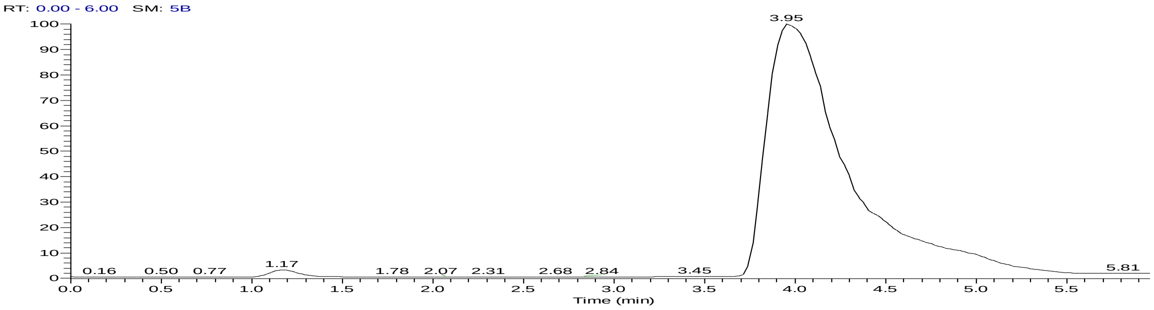

(b)

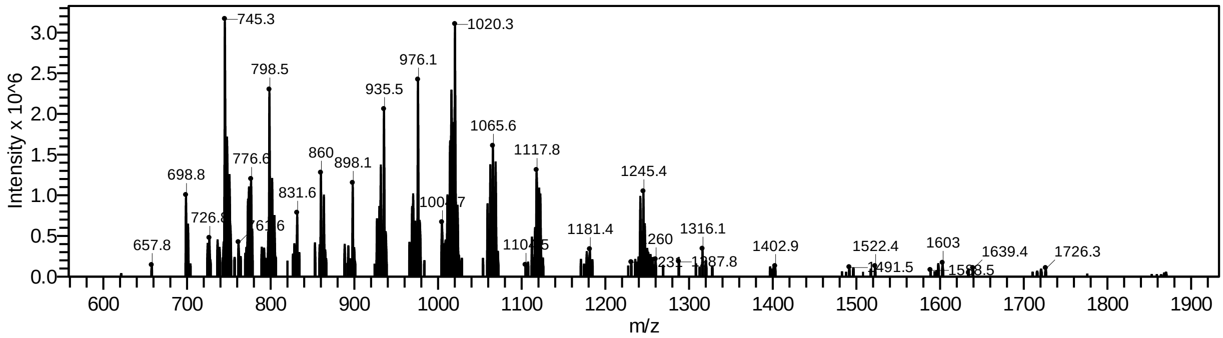

(c)

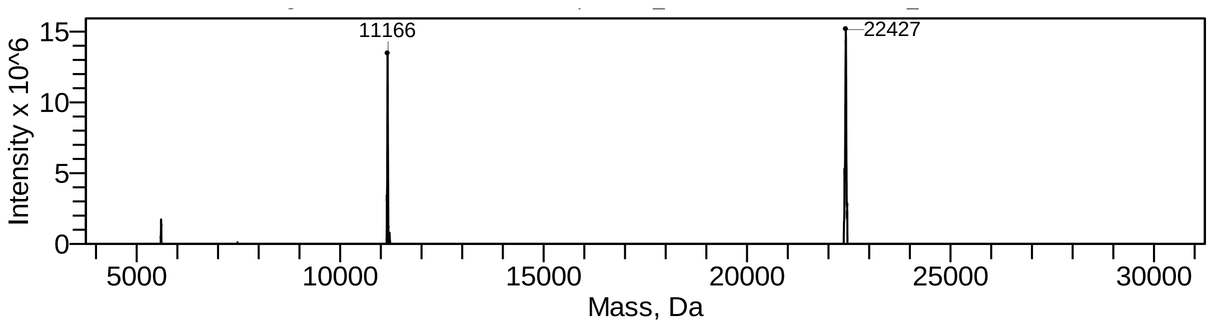

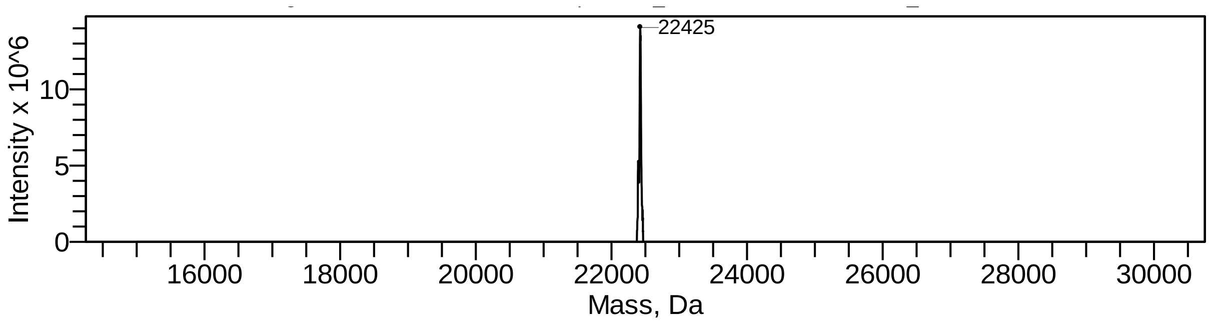

Figure S32: (a) TIC, (b) non-deconvoluted and (c) deconvoluted MS data for UbK63C-pyridazinedione-UbG76C conjugate.

Ubiqutin-pyridazinedione-GFP conjugates

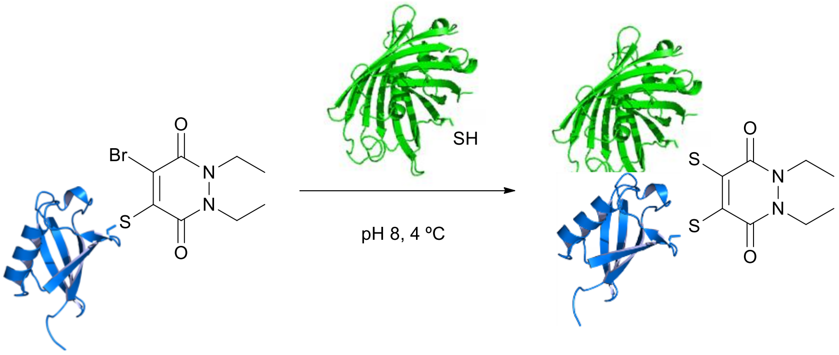

Ubiquitin-pyridazinedione-GFP conjugates were prepared using the following method. The bromopyridazinedione modified ubiqutin was prepared as above, then excess dibromopyridazinedione was removed by ultracentrifugation using VivaSpin sample concentrators (GE Healthcare, 5,000 MWCO). GFP mutant S147C (2.6 mg mL^{-1}, 100 μL) in PBS was buffer exchanged by ultracentrifugation into sodium phosphate buffer pH 8 (50 mM sodium phosphate, 75 mM NaCl, pH 8). Both protein samples were concentrated to half their original volume (to give 50 μL) and mixed together in a 1:1 volume ratio, to give a final volume of 100 μL. This reaction mixture was incubated on ice for 16 h. Analysis using LC-MS showed the coupling was complete.

UbK48C-pyridazinedione-GFPS147C conjugate

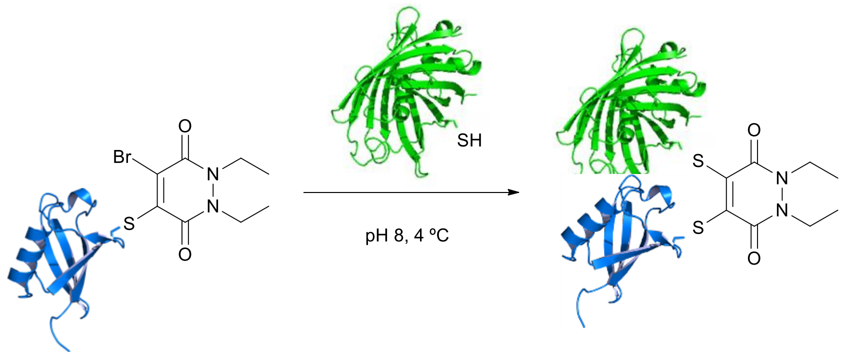

Expected mass: 40,599

Observed mass: 40,605

(a)

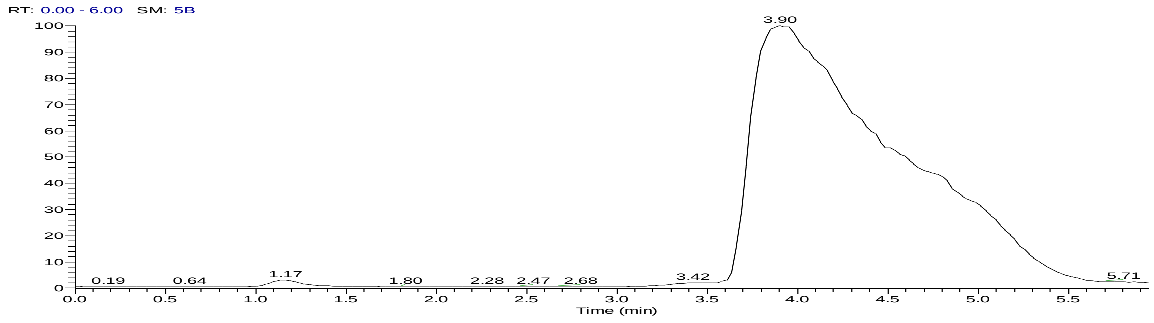

(b)

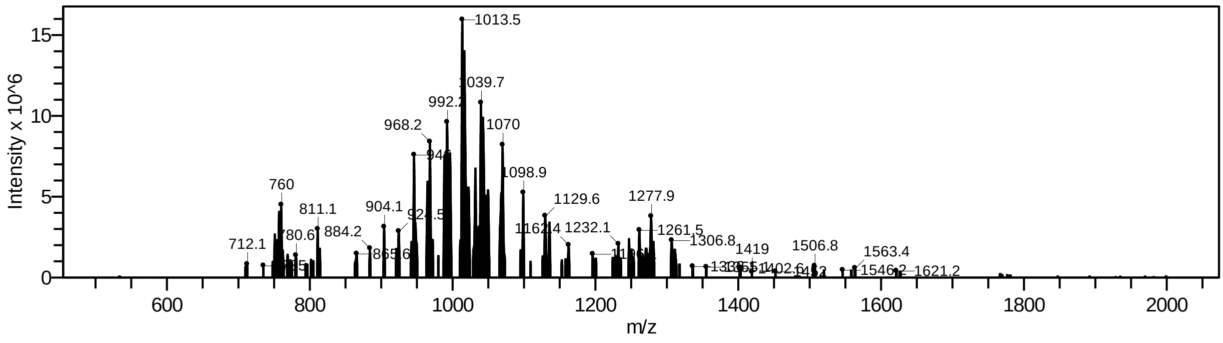

(c)

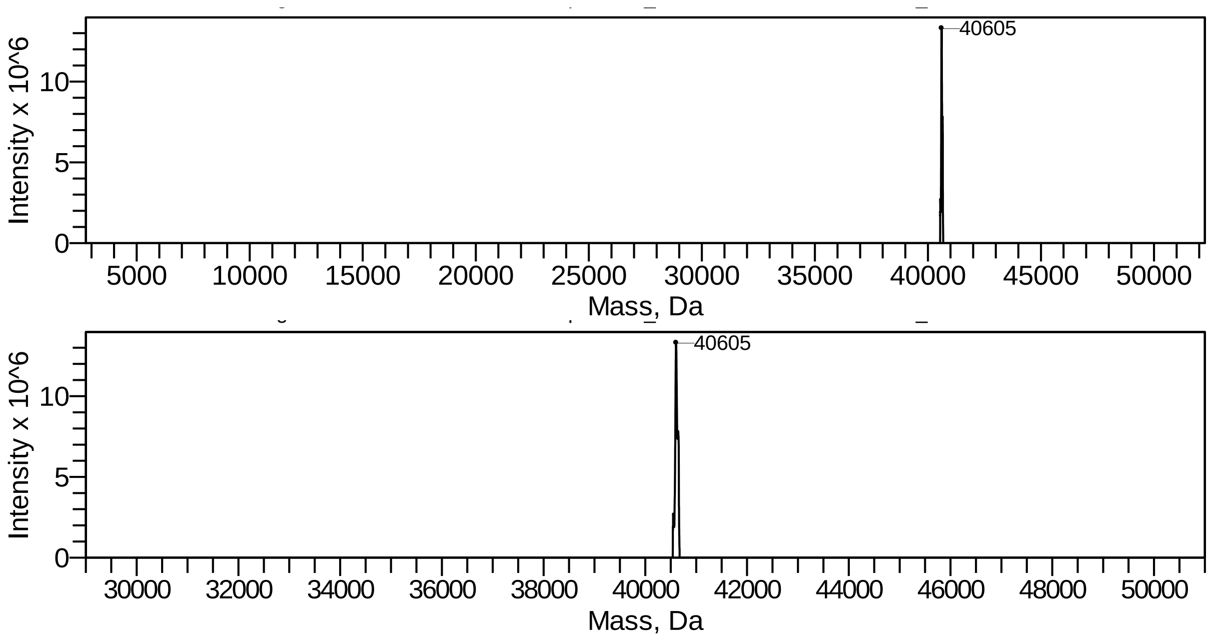

Figure S33: (a) TIC, (b) non-deconvoluted and (c) deconvoluted MS data for UbK48C-pyridazinedione-GFPS147C conjugate.

UbG76C-pyridazinedione-GFPS147C conjugate

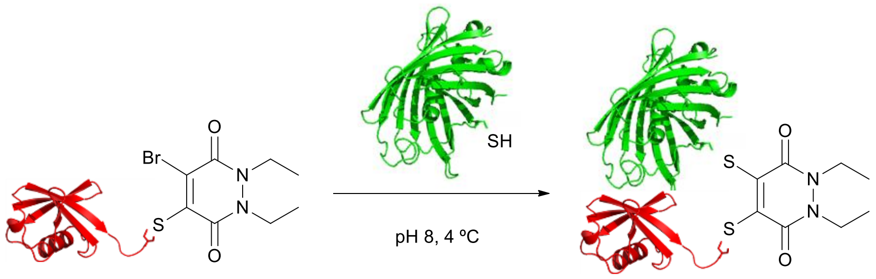

Expected mass: 40,670

Observed mass: 40,676

(a)

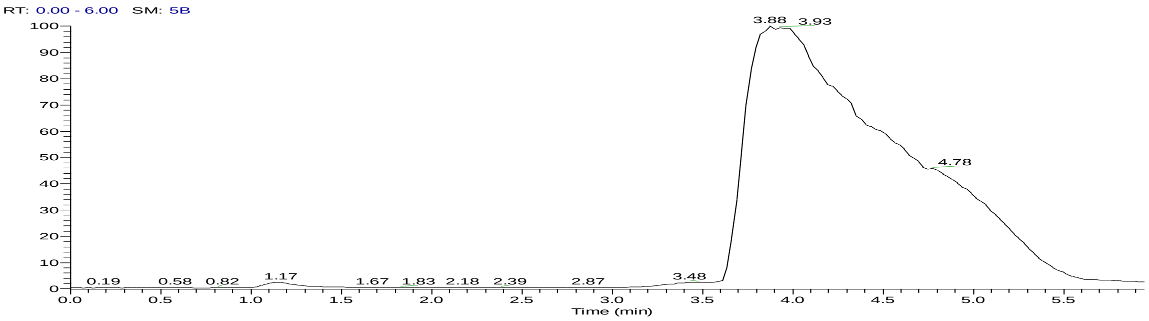

(b)

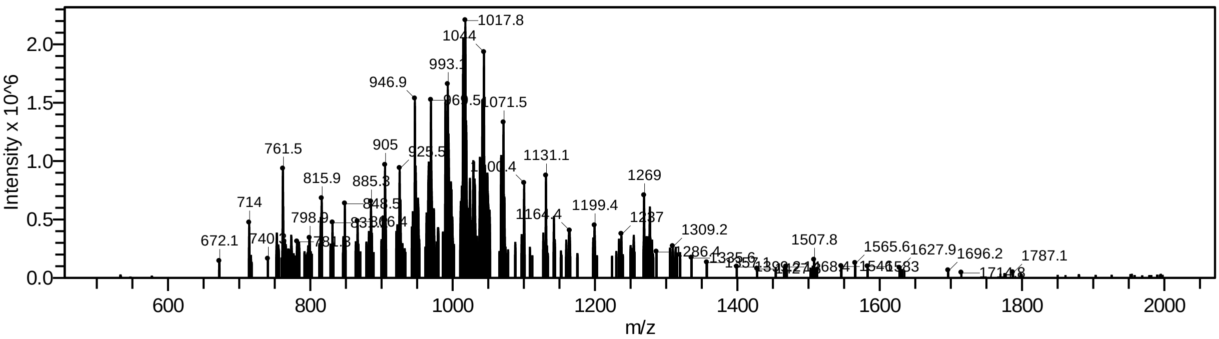

(c)

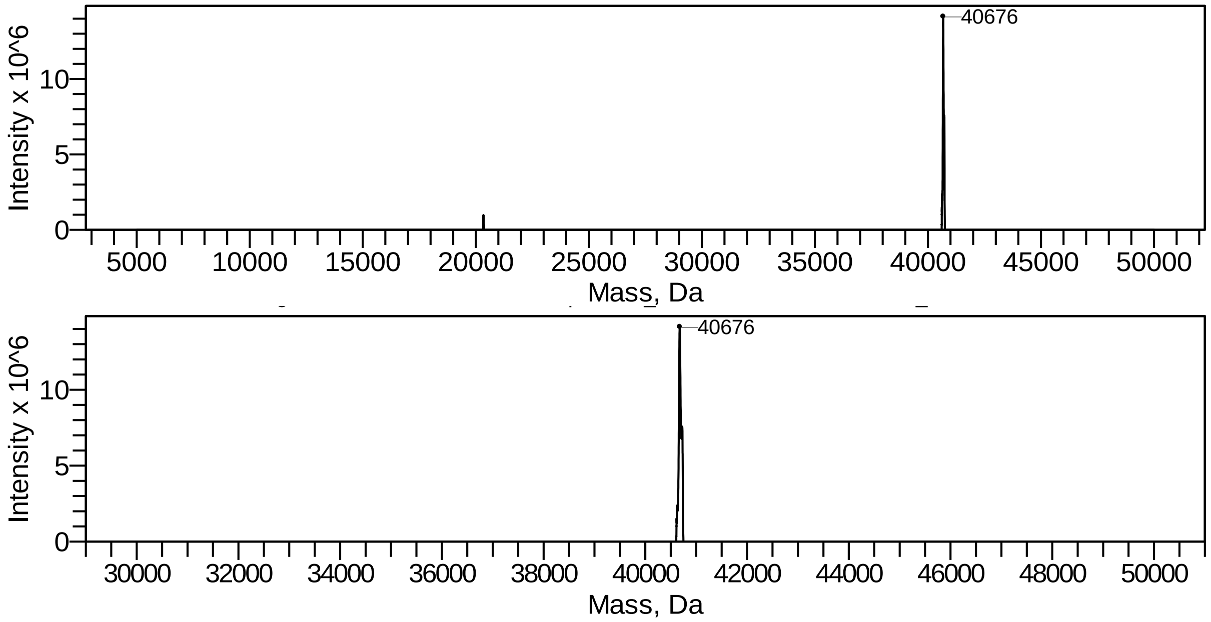

Figure S34: (a) TIC, (b) non-deconvoluted and (c) deconvoluted MS data for UbG76C-pyridazinedione-GFPS147C conjugate.

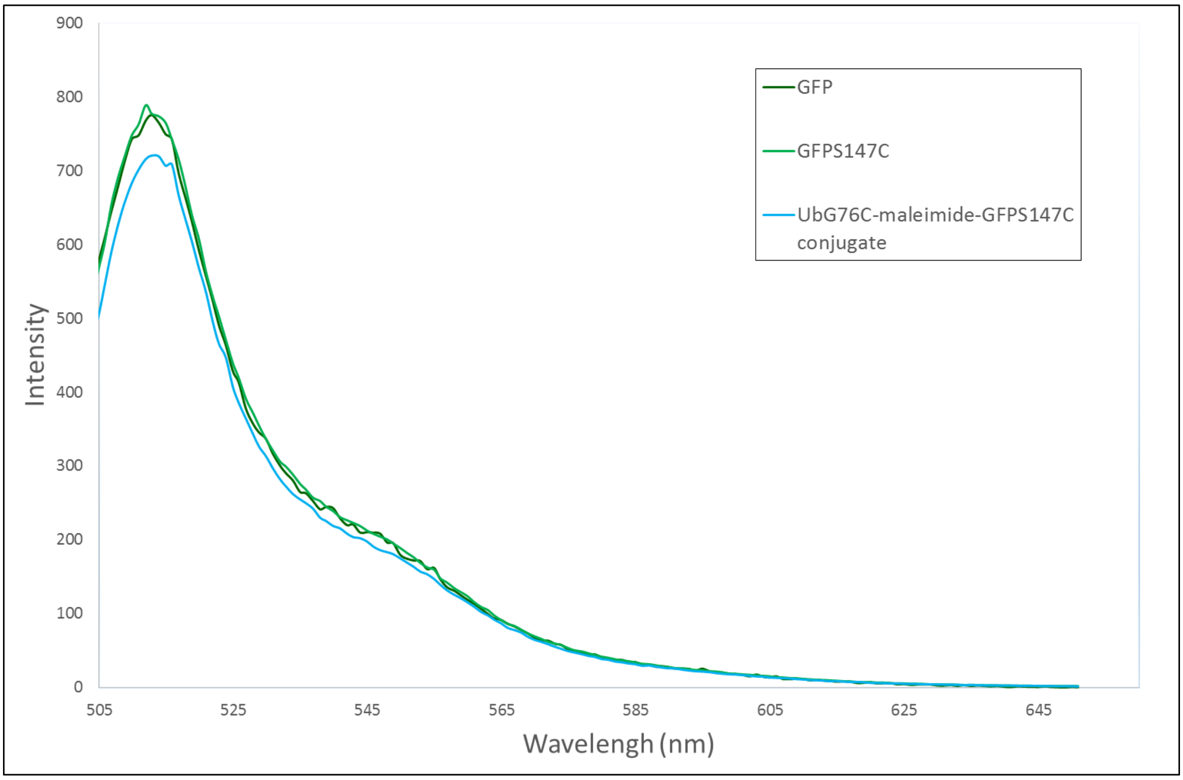

Figure S35: Fluorescence emission spectrum for wild type GFP, GFPS147C and UbG76C-maleimide-GFPS147C upon excitation at 494 nm. Fluorescence measurements were made using a Cary Eclipse fluorescence spectrophotometer.

References

1. V. Chudasama, M. E. Smith, F. F. Schumacher, D. Papaioannou, G. Waksman, J. R. Baker and S. Caddick, *Chem. Commun.*, 2011, **47**, 8781.
2. P. Moody, M. E. B. Smith, C. P. Ryan, V. Chudasama, J. R. Baker, J. Molloy and S. Caddick, *ChemBioChem*, 2012, **13**, 39.
